# Interaction of the Factor H Family Proteins FHR-1 and FHR-5 With DNA and Dead Cells: Implications for the Regulation of Complement Activation and Opsonization

**DOI:** 10.3389/fimmu.2020.01297

**Published:** 2020-07-16

**Authors:** Éva Kárpáti, Alexandra Papp, Andrea E. Schneider, Dávid Hajnal, Marcell Cserhalmi, Ádám I. Csincsi, Barbara Uzonyi, Mihály Józsi

**Affiliations:** ^1^Department of Immunology, ELTE Eötvös Loránd University, Budapest, Hungary; ^2^MTA-ELTE Complement Research Group, Department of Immunology, ELTE Eötvös Loránd University, Budapest, Hungary

**Keywords:** complement, factor H protein family, pentraxin, necrotic cell, DNA, opsonization, CRP, PTX3

## Abstract

Complement plays an essential role in the opsonophagocytic clearance of apoptotic/necrotic cells. Dysregulation of this process may lead to inflammatory and autoimmune diseases. Factor H (FH), a major soluble complement inhibitor, binds to dead cells and inhibits excessive complement activation on their surface, preventing lysis, and the release of intracellular material, including DNA. The FH-related (FHR) proteins share common ligands with FH, due to their homology with this complement regulator, but they lack the domains that mediate the complement inhibitory activity of FH. Because their roles in complement regulation is controversial and incompletely understood, we studied the interaction of FHR-1 and FHR-5 with DNA and dead cells and investigated whether they influence the regulatory role of FH and the complement activation on DNA and dead cells. FH, FHR-1, and FHR-5 bound to both plasmid DNA and human genomic DNA, where both FHR proteins inhibited FH–DNA interaction. The FH cofactor activity was inhibited by FHR-1 and FHR-5 due to the reduced binding of FH to DNA in the presence of the FHRs. Both FHRs caused increased complement activation on DNA. FHR-1 and FHR-5 bound to late apoptotic and necrotic cells and recruited monomeric C-reactive protein and pentraxin 3, and *vice versa*. Interactions of the FHRs with pentraxins resulted in enhanced activation of both the classical and the alternative complement pathways on dead cells when exposed to human serum. Altogether, our results demonstrate that FHR-1 and FHR-5 are competitive inhibitors of FH on DNA; moreover, FHR–pentraxin interactions promote opsonization of dead cells.

## Introduction

The complement system is a key humoral component of innate immunity, and in addition to its many other functions, it is involved in the clearance of waste material, such as immune complexes and apoptotic and necrotic cells (1, 2). Whereas, dying cells are efficiently removed under physiological conditions, defective clearance of dead cells may lead to pathologies and the generation of autoantibodies and thus may be the basis for the development of autoimmune diseases, such as systemic lupus erythematosus, since dying cells are a potential source of self-antigens (3–6). The surface of dying cells is modulated, some molecules that serve as “do not eat me signals” are downregulated, and other ligands that promote phagocytosis are exposed (1, 7).

Dying cells also expose ligands that bind initiator molecules of the various complement pathways, so that complement activation and opsonin deposition on the dead cell surface may enhance phagocytotic clearance (1, 8). The role of C1q and mannose-binding lectin (MBL) in this process is well-documented; these molecules may also interact directly with receptors, such as the calreticulin/CD91 complex, on phagocytes (9). The initiator molecules of the classical (C1q) and lectin pathways (e.g., MBL) can also be recruited by the pentraxins C-reactive protein (CRP) and pentraxin 3 (PTX3), which themselves bind to dying cells via various ligands (8, 10). Notably, these pentraxins may also recruit soluble complement regulators, such as factor H (FH) and C4b-binding protein (C4BP), which in turn limit excessive complement activation on the surface (11–14). Properdin was described to bind to dead cells, and DNA exposed on dying cells was identified as one of the properdin ligands (15, 16). Properdin can bind C3b and activate the alternative complement pathway and also stabilizes the C3bBb alternative pathway C3 convertase enzyme, thereby directing the deposition of C3 fragments to the cell surface and driving the amplification loop (17–19).

Altogether, the activation of the three complement pathways on dead cells results in C3- and/or C4-fragment deposition and enhanced opsonophagocytosis (20). However, even though cell membrane-anchored complement regulators are downregulated on dying cells, further complement activation, such as C5 convertase formation and terminal pathway activation, and the lysis of dying cells are prevented by the serum-derived complement inhibitors FH and C4BP (21–23). This inhibition is essential, because the lack of complement regulation may lead to inflammation and autoimmune processes.

The FH protein family includes FH and FH-like protein 1 (FHL-1), both of which are derived from the *CFH* gene, and five FH-related proteins (FHR-1 to FHR-5) that are derived from the five *CFHR* genes (24–26). These FH family proteins exclusively consist of complement control protein (CCP) domains (also called Sushi domains or short consensus repeats, SCRs). FH is the major soluble regulator of the alternative complement pathway. The complement inhibitory functions of FH (and FHL-1), namely, convertase decay accelerating activity, interference with assembly of the C3bBb convertase through competition with factor B for the binding of C3b, and factor I cofactor activity for the inactivation of C3b, are mediated by the N-terminal CCPs 1–4. All five FHRs lack domains homologous to FH CCPs 1–4; thus, they lack FH-like complement inhibiting activities, although roles in complement regulation have been reported for some of them (27–32). The function of the FHRs is incompletely understood and partly debated; however, recent results demonstrated competition between FHRs and FH for the same ligands causing impaired regulatory activity of FH (24, 33–39). In addition, FHR-1, FHR-4, and FHR-5 were shown to have a direct complement activating function, by binding C3b and allowing formation of the C3bBb alternative pathway C3 convertase (36, 37, 40) or by binding CRP and thus activating the classical pathway (37, 41, 42). The association of *CFH* and *CFHRs* with several complement-mediated diseases strongly supports complement modulating activities of the FHR proteins (24, 25, 43, 44).

FH was shown to bind to Annexin II, DNA, and histones on the surface of apoptotic cells; DNA binding occurs through FH CCPs 6–8, and 19–20 (45). FH can be detected within and on the surface of dead cells, and apoptotic cells are able to internalize it (45, 46). FH colocalizes with genomic DNA (gDNA) intracellularly and with DNA on the surface of apoptotic cells and displays cofactor activity when bound to DNA (45, 46). FH was also shown to bind to extracellular DNA traps (47). Although binding of FHRs to DNA has not yet been analyzed in detail, it was demonstrated that recombinant FHR-2 and FHR-5 bind to necrotic HUVECs and CHO cells (48). A few recent studies indicated that FHR binding to necrotic cells has functional relevance. In the case of necrotic HUVECs, but not on CHO cells, FHR-5 but not FHR-2 was able to increase C3 deposition (48). Furthermore, FHR-1 facilitated the formation of the C3bBb convertase on necrotic cells and enhanced activation of the alternative pathway when necrotic cells were pretreated with monomeric CRP (mCRP) (37). Similarly, the murine FHR protein FHR-B bound to necrotic cells and enhanced C3 deposition (35).

Therefore, the aim of this study was to characterize the interaction of FHR-1 or FHR-5 with DNA and dead cells and investigate how they influence the regulatory role of FH and complement activation.

## Materials and Methods

### Materials

FHR-1, FHR-4A, FHR-4B, and FHR-5 fragments CCPs 3–7, 5–9, and 8–9 were expressed in *Spodoptera frugiperda* (Sf9) cells using the pBSV-8His baculovirus expression vector (49) and purified by nickel affinity chromatography. Recombinant human FHR-5, PTX3, anti-human PTX3, and anti-FHR-5 mAbs were obtained from R&D Systems (Wiesbaden, Germany). Purified human FH, C3b, FI, recombinant human CRP [pentameric (pCRP)], goat anti-factor B, goat anti-C4, goat anti-human FH antisera, anti-Histone H4 pAb, and mouse anti-double-stranded DNA (dsDNA) mAb (clone BV16-13) were purchased from Merck Ltd. (Merck Kft., Budapest, Hungary). The anti-myeloperoxidase (MPO) mAb was purchased from HyTest (Turku, Finland). The anti-FH monoclonal Abs A254 and A255 and the anti-FB mAb were from Quidel (obtained from Biomedica, Budapest, Hungary), and mAb C18 was from Enzo Life Sciences (Farmingdale, New York; obtained through Biomarker, Gödöllő, Hungary). Bovine serum albumin (BSA) was from Applichem (Darmstadt, Germany). Human serum albumin (HSA) and the anti-mCRP mAb were purchased from Sigma-Aldrich Inc. (St. Louis, MO). Horseradish peroxidase (HRP)-conjugated goat anti-human C3 antibody was obtained from MP Biomedicals (Solon, OH). HRP-conjugated rabbit anti-goat immunoglobulins, goat anti-mouse immunoglobulins, and FITC-conjugated anti-C3c antibody were from Dako (Hamburg, Germany). Normal human serum (NHS) was collected from healthy individuals after informed consent. Serum samples were pooled, aliquoted, and kept at −70°C. Alexa 488-conjugated secondary antibodies were from Thermo Fisher Scientific (Budapest, Hungary). mCRP was generated from pCRP as described (50).

### Gel Shift Assays

Gel shift analyses were carried out to visualize the binding of FHR proteins to DNA. Two hundred nanograms linearized pUC57 vector or GeneRuler 1 kb DNA ladder (Thermo Fisher Scientific) was incubated with 10 μg FH or equimolar amounts of FHR proteins in 10 μl final volume in Tris-EDTA buffer (10 mM Tris-HCl, 5 mM EDTA, pH 7.6) for 30 min at 37°C. DNA–protein complexes were separated on 1% agarose gel, and then the DNA was stained with ethidium bromide and visualized using a UV transilluminator.

### Microtiter Plate Binding and Complement Activation Assays

To measure binding of the FHRs to DNA, gDNA was purified from Jurkat cells with the GeneJET gDNA Purification Kit according to the manufacturer's protocol (Thermo Fisher Scientific). gDNA was immobilized at 15 μg/ml on high binding microtiter plates (Greiner) overnight at 4°C. The wells were washed in each step with Dulbecco's PBS (DPBS; Lonza; Biocenter, Szeged, Hungary) containing 0.05% Tween 20. After blocking with 4% BSA in 0.05% Tween 20-containing DPBS at 20°C, FHR-1 (250 nM), FHR-5, or FHR-5 fragments (100 nM) were added in DPBS containing Ca^2+^ and Mg^2+^ for 1 h at 20°C. In some experiments, 250 nM FHR-1 was added together with 20 μg/ml mAb C18 or mAb A255. Binding was detected using polyclonal anti-FH (1:1,000) or polyclonal anti-FHR-5 (1:500), as indicated in each figure, and HRP-conjugated rabbit anti-goat Ig (1:1,000). In a reverse setting, 120 nM FHR-5, FHR-1, and HSA were immobilized, and the remaining free binding sites blocked as above and then incubated with increasing concentrations of gDNA in DPBS containing Ca^2+^ and Mg^2+^ for 1 h at 20°C. The binding of gDNA was detected with serial incubations of anti-dsDNA (1:1,000) and HRP-conjugated goat anti-mouse Ig (1:1,000). TMB Plus substrate (BioLegend) was used to visualize binding, and the absorbance was measured at 450 nm.

In competition assays, FHRs were preincubated with dNTP in the indicated concentrations for 15 min before adding to the wells. FHR-1 and FHR-5 binding was measured as above. To determine the competition between FH and FHRs for DNA, 100 nM FH alone and with FHR-1 or FHR-5 was added to the gDNA-coated wells, and FH binding was measured using the monoclonal anti-FH antibody A254 (5 μg/ml) followed by HRP-conjugated goat anti-mouse Ig (1:750).

To measure complement activation on gDNA, Nunc microtiter plate wells (Thermo Fisher Scientific) were coated with 15 μg/ml gDNA in DPBS overnight at 4°C and, after blocking with 4% BSA in 0.05% Tween 20-containing DPBS at 20°C, 5 or 10% NHS was added with or without 150 nM recombinant FHR-5 or 150–1,200 nM FHR-1 for 30 min at 37°C in DPBS containing Ca^2+^ and Mg^2+^ (Lonza) or, to measure alternative pathway activation only, in DPBS containing 5 mM Mg^2+^-EGTA. After washing with 0.05% Tween 20-containing DPBS, formation of the C3bBb convertase was detected using goat anti-FB (1:2,000) followed by HRP-conjugated rabbit anti-goat Ig (1:1,000), and the deposition of C3 fragments was measured with HRP-conjugated anti-C3 (1:1,500).

### Cofactor Assays and Western Blot Analysis

To analyze the effect of FHRs on FH cofactor activity, microtiter plates were coated with 15 μg/ml gDNA overnight at 4°C; wells were washed, blocked, and incubated with FH and FHR proteins as in the microtiter plate binding assays. After washing, 120 nM C3b and 250 nM FI were added for 1 h at 37°C. Supernatants were subjected to 10% SDS-PAGE under reducing conditions and western blotting. The membrane was blocked with DPBS containing 1% BSA, 4% skimmed milk powder and 0.05% Tween 20. C3b fragments were detected with HRP-conjugated anti-C3 antibody (1:8,000) and by an ECL detection kit (Merck).

### Cells

Jurkat E6.1 T cells (European Collection of Cell Cultures; Salisbury, UK) were cultured in RPMI 1640 (Invitrogen) supplemented with 10% FCS (EuroClone) and 50 μg/ml gentamycin (Lonza); human umbilical vein endothelial cells (HUVECs; Lonza) were cultured in Endothelial Cell Basal Medium-2 (EBM-2) supplemented with 10% FCS, hydrocortisone, growth factors (hFGF, VEGF, R3-IGF-1, and hEGF), ascorbic acid, and heparin (Lonza) and retinal pigment epithelial cells (ARPE-19; ATCC) were cultured in Dulbecco's modified Eagle medium: F12 (DMEM: F12; Lonza) supplemented with 10% FCS and penicillin–streptomycin–amphotericin B mix (Lonza). Apoptosis was induced by treating Jurkat cells with 1 μM staurosporine for 24 h. Necrosis was induced by incubating the cells at 65°C for 30 min.

Human neutrophil granulocytes were isolated from peripheral blood of healthy individuals. The studies were approved by the respective national authority (TUKEB ETT, permission number 838/PI/12). Peripheral mononuclear cells were isolated by Ficoll-Hypaque (Sigma Aldrich) density gradient centrifugation. Red blood cells were removed in two steps: first, dextran sedimentation was performed with Dextran T-500 (Pharmacia Fine Chemicals, Uppsala, Sweden), and then the red blood cells were lysed with hypotonic sodium chloride buffer. Purity of the isolated neutrophils was checked by flow cytometry using anti-CD16 and anti-CD14 antibodies (BD Biosciences, Heidelberg, Germany). Neutrophil purity was >95%.

### Flow Cytometry

Binding of FHR-5 and FHR-1 to live, apoptotic and necrotic cells was measured by incubating 5 × 10^5^ cells/sample with the recombinant proteins in DPBS containing Ca^2+^ and Mg^2+^ (Lonza) for 20 min at 20°C. Binding was detected using monoclonal anti-FHR-5 Ab (5 μg/ml) and goat anti-FH pAb (1:500), respectively, followed by Alexa 488-conjugated secondary antibody (1:500). Early and late apoptotic cells were identified by staining with Annexin V-PE and 7-AAD (Life Technologies) according to the manufacturer's protocol. Necrotic cells were labeled with propidium iodide (1 μg/ml). After each incubation step, cells were washed with DPBS containing 1% FCS.

To measure the effect of dNTP on FHR binding to necrotic cells, 75 nM FHR-5 or 300 nM FHR-1 were incubated in DPBS containing Ca^2+^ and Mg^2+^ with or without 12.5 mM dNTP for 30 min at 20°C. The mixture was added to 5 × 10^5^ necrotic Jurkat cells and incubated for 30 min at 20°C. Cells were then washed and the binding of FHR-5 and FHR-1 was detected as described above.

To measure the interaction of FHRs and pentraxins, necrotic cells were preincubated in DPBS containing Ca^2+^ and Mg^2+^ with either PTX3 or mCRP for 20 min at 20°C, then washed and incubated with FHRs and, after washing, FHR-binding was detected as above. In reverse experiments, necrotic cells were incubated with FHR-5 or FHR-1, then washed followed by incubation with PTX3 or mCRP and, after washing, pentraxin binding was detected using biotinylated anti-PTX3 (5 μg/ml) followed by Alexa 488-conjugated streptavidin (1:500) or anti-mCRP (10 μg/ml) followed by Alexa 488-conjugated goat anti-mouse Ig (1:500).

Complement activation on necrotic Jurkat cells was measured after preincubation with FHRs or pentraxins in RPMI-1640 as indicated for each experiment in the figure legends. Cells were then washed to remove non-bound proteins. To measure classical pathway activation, cells were exposed to 1% NHS in RPMI-1640 for 30 min at 37°C. To measure alternative pathway activation, cells were exposed to 5 or 10% NHS in DPBS containing 5 mM MgCl_2_ and 5 mM EGTA for 30 min at 37°C. Classical pathway activation was detected using anti-human C4 (1:200) followed by Alexa 488-conjugated rabbit anti-goat Ig (1:200) and FITC-conjugated anti-C3c (1:200), and AP activation was measured using FITC-conjugated anti-C3c (1:200) and anti-FB (1:200) followed by Alexa 488-conjugated rabbit anti-goat Ig (1:200).

In each experiment, antibodies were added for 15 min on ice and samples were kept in dark. In each sample, 10,000 cells were measured using a FACSCalibur flow cytometer (BD Biosciences, San Jose, CA, USA). Data were analyzed using FCS Express Version 3 software (*De Novo* Software, Los Angeles, CA, USA).

### Binding of Serum-Derived FHR-5 and FHR-1 to Necrotic Cells

2 × 10^6^ necrotic ARPE-19, HUVEC and Jurkat cells were incubated in DPBS containing Ca^2+^ and Mg^2+^ with 50% NHS for 30 min at 37°C. Cells were thoroughly washed, then lysed with buffer containing cOmplete™ Protease Inhibitor Cocktail (Roche) supplemented with 1% Triton X-100 (Sigma-Aldrich). Cell lysates were centrifuged at 10,000 *g* for 10 min, separated by 10% SDS-PAGE, transferred to nitrocellulose membrane and developed with anti-FHR-5 (1:500) and anti-FH (1:5,000) pAbs, respectively, using HRP-conjugated rabbit anti-goat Ig (1:5,000) and an ECL detection kit (Merck).

### Confocal Laser Scanning Microscopy

To measure colocalization between FHR-5 or FHR-1 and DNA, 5 × 10^5^ necrotic HUVEC were treated with 300 nM FHR-5 or FHR-1 in DPBS containing Ca^2+^ and Mg^2+^ for 30 min at 20°C and, after washing, labeled with monoclonal anti-FHR-5 (5 μg/ml) or goat anti-FH (1:500) for 15 min at 4°C, followed by washing and incubation with the corresponding Alexa 488-conjugated secondary antibodies (1:500). After washing, gDNA was labeled with 1 μg/ml propidium iodide.

To visualize FHR-5 and FHR-1 binding to neutrophil extracellular traps (NETs), 5 × 10^5^ neutrophils were allowed to adhere to Nunc Lab-Tek borosilicate chambered cover glass microplates (Thermo Fisher Scientific), then NETs were induced with PMA and stained with Sytox Orange (Molecular Probes-Invitrogen) as previously described (51). To confirm NET formation, in addition to DNA parallel samples were stained with anti-Histone H4 or anti-MPO (both 1:500) and Alexa 488-conjugated goat anti-rabbit Ig (1:1,000). To analyze FHR-5 and FHR-1 binding, NETs were prepared and incubated with 300 nM FHR-5 or FHR-1 in DPBS at 20°C for 20 min. After careful washing with DPBS, goat anti-FHR-5 antibody (1:500) or goat anti-FH antiserum (1:500) was added, followed by incubation with Alexa 488-labeled anti-goat Ig (1:500) at 20°C in the dark for 30 min.

Fluorescence microscopy was carried out with a FluoView 500 confocal laser-scanning microscope (Olympus Europe, Hamburg, Germany), equipped with argon-ion laser (488 nm) and two He-Ne lasers (with 543 and 632 nm excitation wavelengths). Fluorescence images (1,024 × 1,024 pixels) were acquired using a 60× oil-immersion objective. Images were processed by ImageJ software (http://rsbweb.nih.gov/ij, National Institutes of Health, Bethesda, MD, USA).

### Statistical Analysis

Statistical analysis was performed using GraphPad Prism version 5.00 for Windows (GraphPad Software, San Diego, California). A *p* < 0.05 was considered statistically significant.

## Results

### FHR-5 and FHR-1 Bind to DNA and Neutrophil Extracellular Traps (NETs)

**Figure 1:**
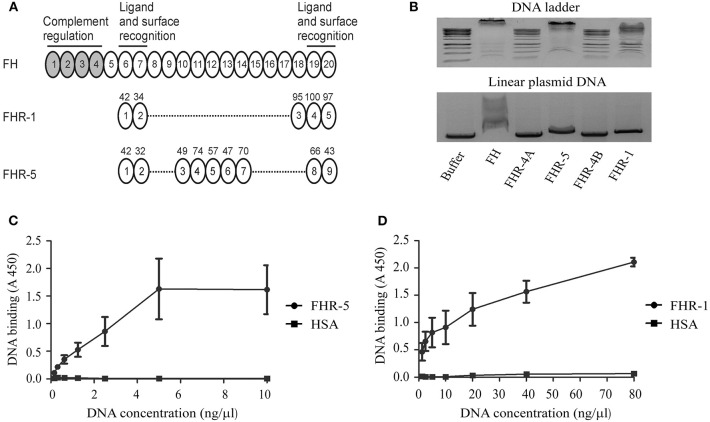
FHR-1 and FHR-5 bind to DNA. **(A)** The schematic drawing shows FH, which is composed of 20 CCP domains, of which CCPs 1–4 mediate the complement regulatory activity of the protein and CCPs 6–7 and 19–20 mediate ligand and surface binding. The CCP domains of FHR-1 and FHR-5 are shown aligned with the homologous FH domains; the numbers above the domains indicate the amino acid sequence identity (in %) with the corresponding FH domain. **(B)** Gel shift assays were performed to analyze the interaction of FH family proteins with DNA in the fluid phase. DNA ladder or linearized plasmid DNA were incubated with 10 μg FH and equimolar amounts of the recombinant FHR proteins, FH being a positive control, as indicated, separated on agarose gel, and visualized by ethidium bromide staining. FHR-4A, which is also composed of nine CCPs and is similar in size to FHR-5, and FHR-4B, which is composed of five CCPs and is similar in size to FHR-1, were used as additional controls. DNA retention was observed in the case of FH, FHR-1, and FHR-5. Images are representative of three experiments. ELISA was carried out to confirm DNA binding of FHR-5 and FHR-1. To this end, 120 nM FHR-5 **(C)**, FHR-1 **(D)**, and HSA as a negative control were immobilized, and after blocking, gDNA isolated from Jurkat cells was added in increasing concentrations. gDNA binding was detected using an anti-dsDNA mAb. Data are means ± SD derived from three independent experiments.

FHR-1 and FHR-5 were previously shown to bind to FH ligands, such as pentraxins (13, 31, 36, 37, 43), due to their structural homology (Figure 1A). Because FH interacts with DNA through CCP domains 6–8 and 19–20 (45), we investigated whether these FHR proteins can also bind to DNA. To this end, recombinant human FHR-1 and FHR-5 were incubated in equimolar amounts with linearized plasmid DNA or DNA ladder in a gel-shift assay. FH was used as a positive control, and recombinant FHR-4B and FHR-4A, which are similar in size to FHR-1 and FHR-5, respectively, but differ from them in domain composition, were also analyzed. Binding to DNA was observed in the case of FH, FHR-1 and FHR-5 at pH 7.4 and 37°C, whereas no DNA binding was detected in the case of FHR-4A and FHR-4B under these conditions (Figure 1B).

To further analyze DNA binding and confirm the interaction of FHR-1 and FHR-5 also with human gDNA, ELISA was performed. When equimolar amounts of FHR-5 and FHR-1 were immobilized on microplate wells and gDNA isolated from Jurkat T cells was added in increasing concentrations, we detected a dose-dependent binding of the DNA to both FHRs using an anti-dsDNA Ab (Figures 1C,D). Binding of gDNA to FHR-5 reached saturation at 5 ng/μl DNA concentration, whereas in the case of FHR-1 an ~8-fold higher DNA concentration was required to reach similar binding, and the maximal DNA binding required even higher concentrations under the experimental conditions (Figures 1C,D).

**Figure 2:**
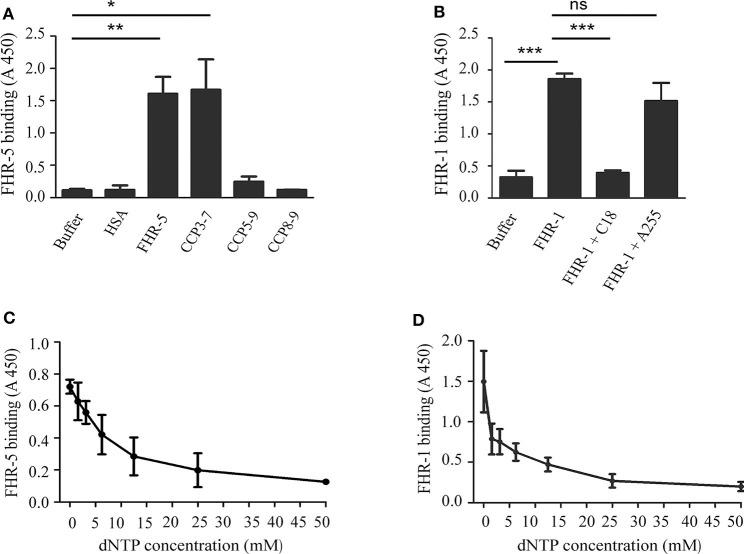
Characterization of the interaction of FHR-5 and FHR-1 with DNA. ELISA was carried out to determine the DNA binding site in FHR-5 and in FHR-1. **(A)** gDNA was immobilized and, after blocking, incubated with FHR-5, FHR-5 fragments, and HSA as negative control. Binding was detected with polyclonal anti-FHR-5 Ab. **(B)** gDNA was immobilized and, after blocking, incubated with FHR-1 alone or in the presence of the C-terminally binding mAb C18 and an indifferent mAb A255. Binding of FHR-5 **(C)** and FHR-1 **(D)** to gDNA is dose-dependently inhibited by dNTP. gDNA was immobilized and FHR-5 and FHR-1, which were preincubated with increasing concentrations of dNTP, were added. FHR5 binding was detected with polyclonal anti-FHR-5, and FHR-1 binding was detected with polyclonal anti-FH. Data are means ± SD derived from three **(A,B)** or four **(C,D)** independent experiments, *p < 0.05, **p < 0.01, ***p < 0.001, one-way ANOVA; ns, not significant.

To determine the DNA binding site within FHR-5, FHR-5 fragments (CCP3-7, CCP5-9, and CCP8-9) were generated and expressed in insect cells, purified, and used in ELISA. Microtiter plates were coated with gDNA, then incubated with equimolar amounts of the FHR-5 fragments. A prominent binding to gDNA was detected only in the case of the CCP3-7 fragment, suggesting that the DNA binding site of FHR-5 is localized in these domains (Figure 2A). In the case of FHR-1, we investigated whether the DNA binding site is located in the C-terminal FHR-1 domains that are homologous to the DNA-binding FH CCPs 19 and 20. To this end, we used the mAb C18, which binds to CCP20 of FH and also to CCP5 of FHR-1, which has a high (97%) sequence identity with FH CCP20 (52, 53). The mAb C18 completely blocked the binding of FHR-1 to gDNA, whereas the control A255 mAb had no effect (Figure 2B).

To confirm the specificity of DNA binding, FHR-5 and FHR-1 were preincubated with soluble dNTP in increasing concentrations and then the mixtures were added to gDNA, which was immobilized in microplate wells. In both cases, the preincubation with dNTP decreased FHR binding to gDNA in a dose-dependent manner (Figures 2C,D).

In addition, to study interaction with DNA released through a more natural cellular process, we investigated whether these two FHR proteins are also able to associate with NETs. We used a PMA-induced NET model (51, 54) and, after DNA staining, FHR-1 or FHR-5 were added and then detected with the corresponding antibodies. On confocal images we observed binding of both proteins that showed colocalization with DNA in NETs (Supplementary Figure 1).

### FHR-5 and FHR-1 Compete With FH for Binding to gDNA and Inhibit FH Cofactor Activity

**Figure 3:**
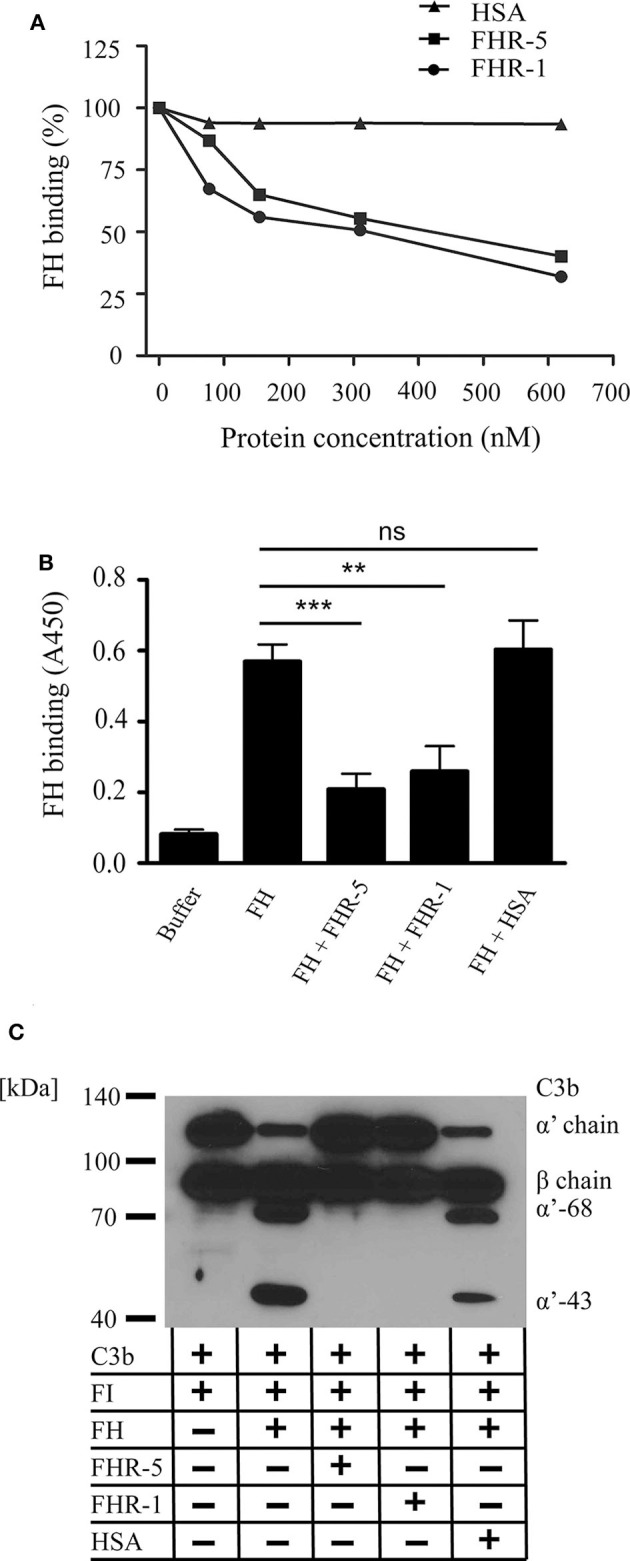
FHR-5 and FHR-1 compete with FH on gDNA and reduce FH cofactor activity. **(A)** FHR-5 and FHR-1 dose-dependently inhibit FH binding to gDNA. FH (100 nM) was added to immobilized gDNA in the presence of increasing concentrations of FHR-5, FHR-1, or HSA. FH binding was detected with the FH-specific mAb A254 that does not recognize FHR-5 or FHR-1. A representative experiment is shown. **(B)** Inhibition experiments were repeated at 300 nM FHR-5 and FHR-1. Data are means ± SD derived from four independent experiments. **p < 0.01, ***p < 0.001, ns, not significant, one-way ANOVA. **(C)** Analysis of the cofactor activity of DNA-bound FH in the presence of FHR-1 or FHR-5. gDNA was immobilized, 100 nM FH was added alone or in the presence of 300 nM FHR-5, FHR-1, or HSA as negative control. After washing, C3b and FI were added, and the plate was incubated at 37°C for 60 min. Supernatants were analyzed by SDS-PAGE and western blot for C3b cleavage products under reducing conditions. Inhibition of FH binding to gDNA by FHR-1 and FHR-5 resulted in loss of C3b cleavage. A representative blot from three independent experiments is shown.

Recent reports demonstrated that some of the FHR proteins can act as competitive inhibitors of FH for binding to ligands such as C3b, components of the extracellular matrix, PTX3 and mCRP (33, 36–38). Therefore, we investigated whether FHR-1 and FHR-5 compete with FH for binding to gDNA and also its functional consequence. To this end, FH binding to immobilized gDNA was measured, using a FH-specific monoclonal Ab, in the absence or presence of equimolar amounts of FHR-1, FHR-5 and the negative control protein HSA. Both FHR-1 and FHR-5 significantly inhibited FH binding to DNA in a dose-dependent manner, whereas HSA had no effect (Figures 3A,B).

To analyze the cofactor activity of gDNA-bound FH, first FH was added to immobilized gDNA in the absence or presence of FHR-5, FHR-1 or the negative control protein HSA. After washing, C3b and FI were added for 1 h, then the supernatants were analyzed for C3b fragments by Western blot. FH acted as a cofactor for the FI-mediated cleavage and inactivation of C3b when bound to gDNA, and the inhibition of FH binding to DNA by FHR-5 and FHR-1 resulted in the lack of C3b cleavage (Figure 3C).

### FHR-5 and FHR-1 Enhance Complement Activation on gDNA

**Figure 4:**
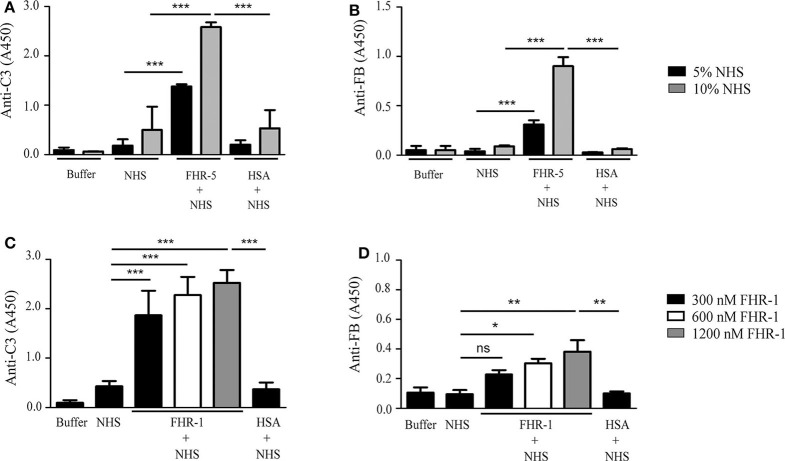
FHR-5 and FHR-1 enhance complement activation on gDNA. gDNA was immobilized in microplate wells and incubated with NHS without or with the addition of 150 nM FHR-5, FHR-1, and HSA as a negative control, at 37°C for 30 min. C3 deposition was detected with anti-C3 Ab, and C3bBb convertase formation was detected with anti-FB Ab. In Mg^2+^-EGTA-containing buffer, which allows alternative pathway activation only, addition of 150 nM FHR-5 to NHS led to increased C3 deposition **(A)** and formation of the C3bBb convertase **(B)**. In contrast to this, 150 nM FHR-1 had no effect on either C3 deposition or C3bBb formation (data not shown), but FHR-1 dose-dependently enhanced C3 deposition **(C)** and, at higher concentrations, C3bBb formation **(D)** in 5% NHS. Data are means ± SD derived from four independent experiments. *p < 0.05, **p < 0.01, ***p < 0.001, ns, not significant, one-way ANOVA. Note the different scales in the *y*-axes.

Both FHR-5 and FHR-1 were shown to support the formation of the alternative pathway C3bBb convertase by binding C3b (36, 37). Therefore, we analyzed whether the FHR-5/FHR-1—DNA interaction can influence C3 fragment deposition and the formation of the C3bBb convertase. To this end, gDNA was immobilized in microplate wells and exposed to 5 or 10% NHS that was supplemented or not with 150 nM FHR-5 in buffer containing 5 mM Mg^2+^-EGTA, which allows for alternative pathway activation only. FHR-5 enhanced both C3 fragment deposition and the formation of the C3bBb convertase, the latter detected by measuring the bound Bb fragment with an anti-FB antibody, especially at higher serum concentration (Figures 4A,B). By contrast, FHR-1 in 150 nM (data not shown) and 300 nM concentrations had no effect on C3bBb formation on gDNA exposed to 5% serum in Mg^2+^-EGTA containing buffer, but significantly enhanced deposition of both C3 fragments and Bb at higher FHR-1 concentrations (Figures 4C,D).

### FHR-5 and FHR-1 Bind to Apoptotic and Necrotic Cells

**Figure 5:**
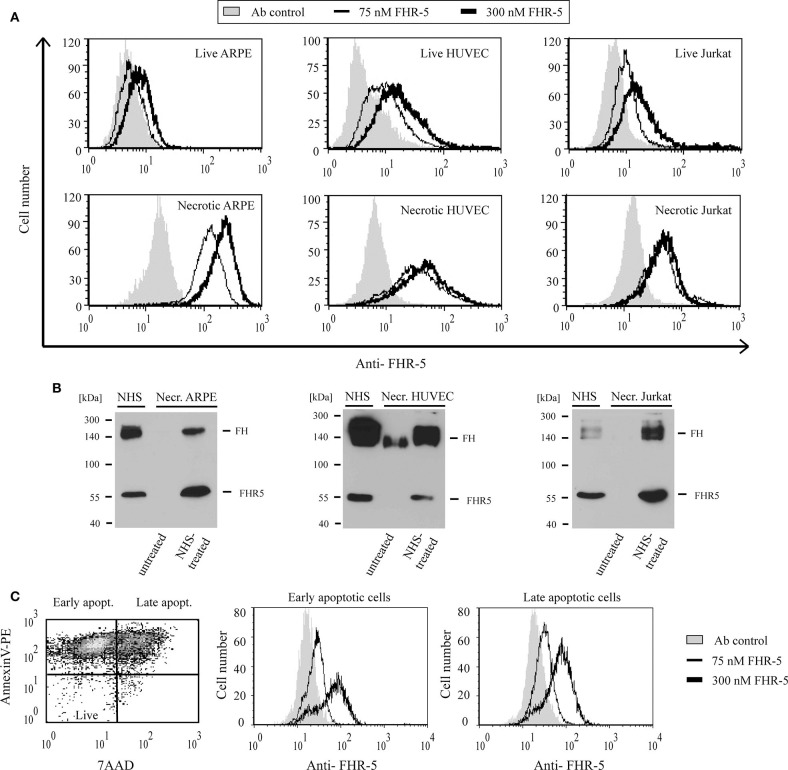
FHR-5 binds to live, apoptotic, and necrotic cells. **(A)** Binding of 75 nM (thin lines) and 300 nM (thick lines) recombinant FHR-5 to live and necrotic retinal pigment epithelial cells (ARPE-19), human umbilical vein endothelial cells (HUVECs), and Jurkat T cells in DPBS with Ca^2+^/Mg^2+^ was measured by flow cytometry using monoclonal anti-FHR-5 and a corresponding secondary Ab. Representative data of three experiments are shown. Gray histograms indicate antibody binding controls without FHR-5. **(B)** Binding of native FHR-5 from NHS to necrotic cells. Necrotic cells were incubated with NHS, washed, and then lysed. Cell lysates were analyzed by SDS-PAGE and western blot using polyclonal anti-FHR-5 Ab. Representative blots of three experiments are shown. **(C)** FHR-5 binding to apoptotic cells was measured on staurosporine-treated Jurkat T cells. The dot plot (left panel) shows the ratio of early and late apoptotic Jurkat cells after 1-day staurosporine treatment and labeling with Annexin V-PE and 7-AAD. After gating, histograms show binding of 75 nM FHR-5 (thin lines) and 300 nM FHR-5 (thick lines) to early (middle panel) and late (right panel) apoptotic Jurkat cells. Representative data of three experiments are shown.

Because of the high degree of sequence identity (36–100%) between the two C-terminal CCP domains in FHRs and the homologous CCPs 19–20 of FH, which harbors a major cell surface recognition site, we analyzed whether FHR-5 and FHR-1 were able to bind to diverse cell lines (ARPE-19, HUVEC, Jurkat) in different states (live, necrotic, apoptotic). Binding of the FHRs to the cells was measured by flow cytometry using recombinant proteins. FHR-5 bound to the three tested live, necrotic, early apoptotic and late apoptotic cells in a dose-dependent manner, although the intensity of binding differed (Figures 5A,C). FHR-5 bound to the surface of all necrotic cell lines with higher intensity than to live cells (Figure 5A). To confirm these results with the native protein, necrotic cells were incubated in buffer containing 50% NHS and, after thorough washing, the cells were lysed and FHR-5 binding was analyzed by Western blot. With this approach, binding of serum-derived FHR-5 to necrotic cells could also be detected (Figure 5B).

**Figure 6:**
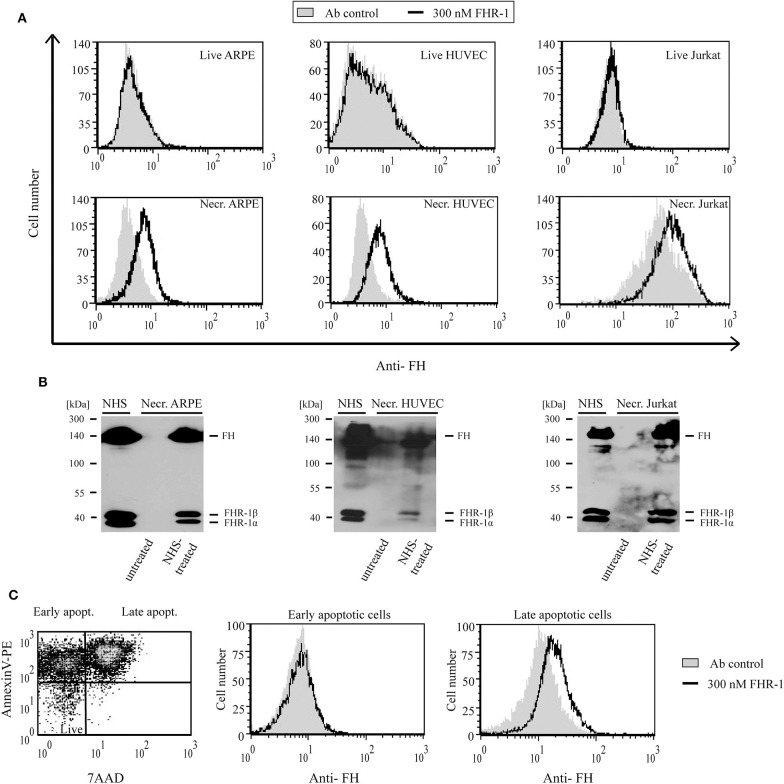
FHR-1 binds to apoptotic and necrotic cells. **(A)** Binding of 300 nM (black lines) recombinant FHR-1 to live and necrotic retinal pigment epithelial cells (ARPE-19), human umbilical vein endothelial cells (HUVECs), and Jurkat T cells in DPBS with Ca^2+^/Mg^2+^ was measured by flow cytometry using polyclonal anti-FH Ab and a corresponding secondary Ab. Gray histograms indicate antibody binding controls without FHR-1. **(B)** Binding of native FHR-1 from NHS to necrotic cells. Necrotic cells were incubated with NHS, washed, and then lysed. Cell lysates were analyzed by SDS-PAGE and western blot using polyclonal anti-FH Ab. Representative blots of three experiments are shown. **(C)** FHR-1 binding to apoptotic cells was measured on staurosporine-treated Jurkat T cells. The dot plot (left panel) shows the ratio of early and late apoptotic Jurkat cells after 1-day staurosporine treatment and labeling with Annexin V-PE and 7-AAD. After gating, histograms show 300 nM FHR-1 binding to early (middle panel) and late (right panel) apoptotic Jurkat T cells. Representative data of three experiments are shown.

In contrast to FHR-5, recombinant FHR-1 did not bind to live ARPE-19, HUVEC or Jurkat cells even at 300 nM concentration; however, when the cells were rendered necrotic, FHR-1 binding was detected (Figure 6A). Similar to FHR-5, binding of native, NHS-derived FHR-1 to all three necrotic cells could be detected by Western blot (Figure 6B). After 1 μM staurosporine treatment of Jurkat cells, only the Annexin V and 7-AAD positive late apoptotic cell population showed FHR-1 binding (Figure 6C).

To measure the possible contribution of DNA in binding of these FHRs to necrotic cells, FHR-5 and FHR-1 were preincubated with dNTP before adding them to necrotic Jurkat cells; this resulted in reduced binding (Supplementary Figures 2A,B). In addition, partial colocalization of FHR-5 and FHR-1 with DNA was found when analyzing necrotic cells with confocal laser scanning microscopy (Supplementary Figure 2C).

### FHR-5 and FHR-1 Enhance Pentraxin Binding, and Inversely, mCRP and PTX3 Enhance FHR-5, and FHR-1 Binding to Necrotic HUVEC

**Figure 7:**
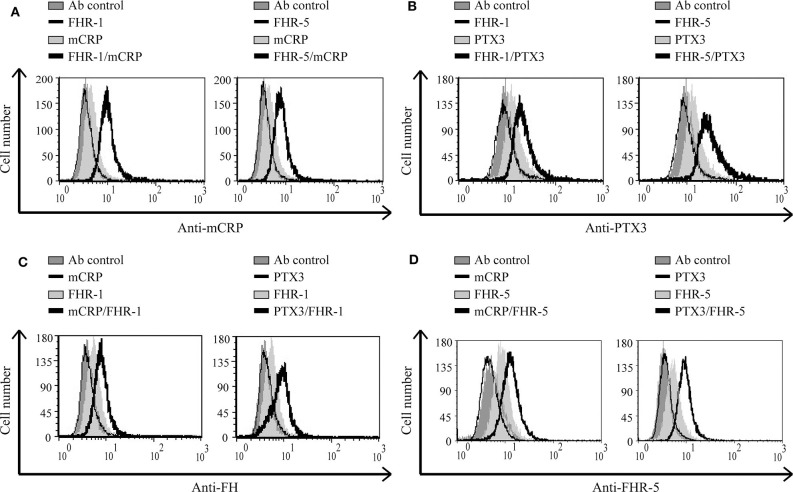
FHR-1 and FHR-5 enhance pentraxin binding and *vice versa* on necrotic cells. Necrotic HUVECs were incubated with 500 nM FHR-1 or 300 nM FHR-5; then 1 μg/ml mCRP or 2.5 μg/ml PTX3 was added. Pentraxin binding was measured by flow cytometry using anti-mCRP **(A)** and anti-PTX3 **(B)** Abs, and the corresponding secondary Abs. In a reverse setting, cells were incubated with 2.5 μg/ml mCRP or PTX3, then 300 nM FHR-1 or 75 nM FHR-5 was added, and their binding was measured using anti-FH **(C)** or anti-FHR-5 **(D)** Abs and the corresponding Alexa 488-labeled secondary Ab. Representative data from three independent experiments are shown.

FHR-1 and FHR-5 were previously shown to interact with pentraxins (13, 31, 36, 37); the mCRP and PTX3 binding sites are in the C-terminal domains of FHR-1 (36, 37). For FHR-5, the CRP-binding was shown to be mediated by CCP3-7 (31), which we could confirm and determined similar binding site for PTX3 (Supplementary Figure 3). We investigated whether FHR-1 and FHR-5 can recruit the pentraxins mCRP or PTX3 to the necrotic cell surface. To this end, necrotic HUVEC cells were pretreated or not with 500 nM FHR-1 or 300 nM FHR-5, then the cells were incubated with pentraxins. Binding of mCRP and PTX3 was detected with the corresponding Abs. The extent of pentraxin binding increased when the necrotic cells were preincubated with FHR-1 or FHR-5, suggesting a cooperation between these molecules (Figures 7A,B). In addition, in a reverse setting when necrotic cells were incubated first with the pentraxins followed by the FHRs, the binding of both FHR-1 and FHR-5 increased, thus mCRP and PTX3 were able to recruit FHR-1 and FHR-5 to necrotic cells (Figures 7C,D).

### Interaction of FHR-5 and FHR-1 With Pentraxins Enhances Complement Activation on Necrotic HUVEC

**Figure 8:**
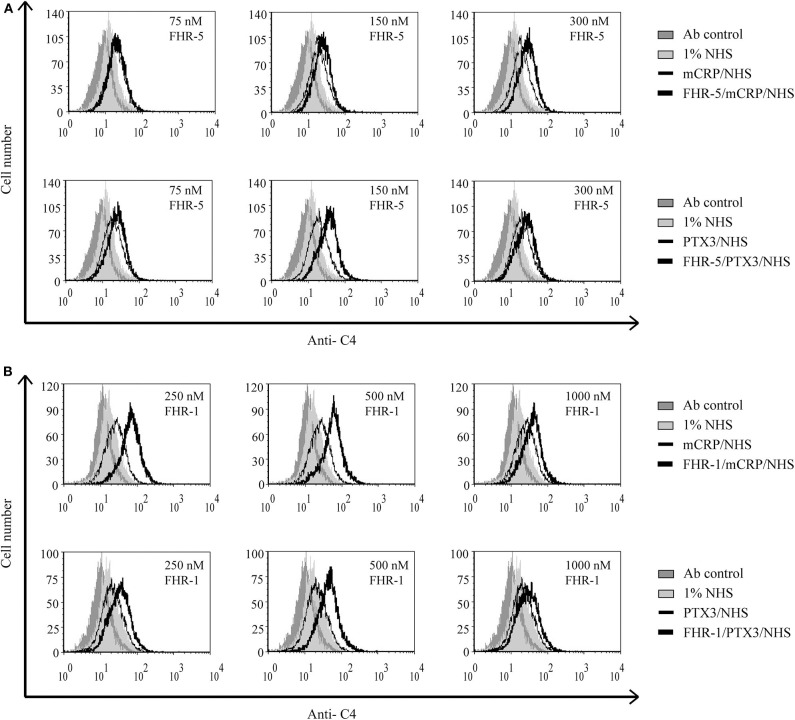
Interaction of FHR-5 and FHR-1 with pentraxins enhances C4 deposition. Necrotic HUVECs were preincubated with increasing amounts of FHR-5 **(A)** or FHR-1 **(B)** and then incubated with 2.5 μg/ml PTX3 or mCRP and exposed to 1% NHS for 30 min at 37°C. C4 deposition was detected by flow cytometry using polyclonal anti-C4 and the corresponding Alexa 488-labeled secondary Ab. Representative data from three independent experiments are shown.

**Figure 9:**
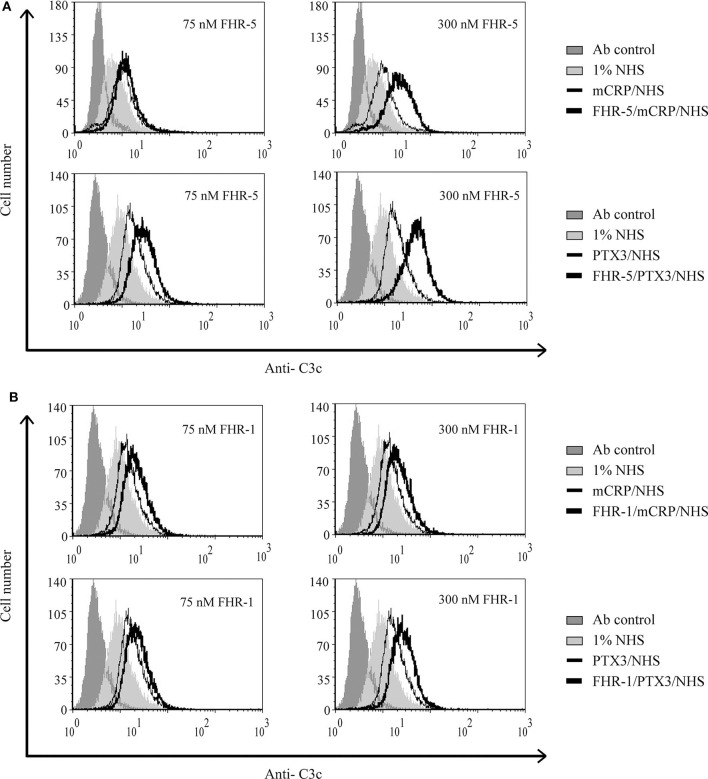
Interaction of FHR-5 and FHR-1 with pentraxins enhances C3 deposition. Necrotic HUVECs were preincubated with increasing amounts of FHR-5 **(A)** or FHR-1 **(B)** and then incubated with 5 μg/ml mCRP or 2.5 μg/ml PTX3 and exposed to 1% NHS for 30 min at 37°C. C3 deposition was detected by flow cytometry using FITC-labeled polyclonal anti-C3c. Representative data from three independent experiments are shown.

**Figure 10:**
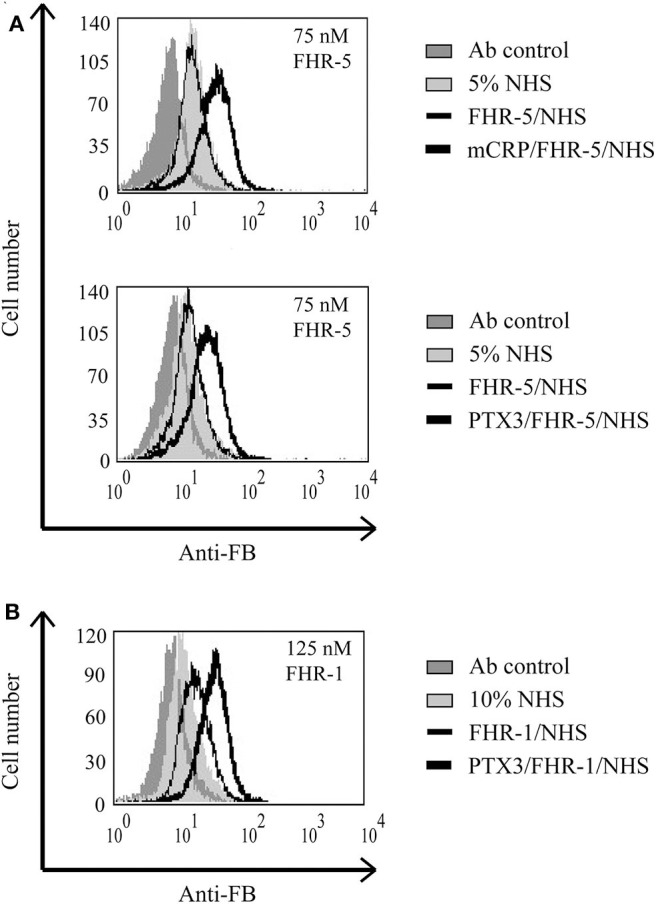
Interaction of FHR-5 and FHR-1 with pentraxins enhances C3bBb formation. Necrotic HUVECs were preincubated with 2.5 μg/ml mCRP or PTX3 and then incubated with 75 nM FHR-5 **(A)** or 125 nM FHR-1 **(B)** and exposed to 5 or 10% NHS in Mg^2+^-EGTA-containing buffer for 30 min at 37°C. Formation of the C3bBb convertase was detected by flow cytometry using polyclonal anti-FB and Alexa 488-labeled secondary Ab. Representative data from three independent experiments are shown.

Recently, we showed that interaction between FHR-1 and mCRP causes increased alternative pathway activation, measured as formation of C3bBb, on necrotic cells (37). Based on the observed reciprocal recruitment of FHRs and pentraxins (Figure 7), we investigated how these interactions can influence complement activation on necrotic cells. First, necrotic HUVEC were preincubated with FHR-5 or FHR-1, followed by incubation with mCRP or PTX3. Then the cells were exposed to 1% NHS. FHR-pretreatment resulted in increased deposition of C4- and C3 fragments, measured with anti-C4 (Figure 8) and anti-C3c (Figure 9), respectively, indicating increased classical pathway activation due to cooperation of these FHRs with pentraxins. In the reverse setting, necrotic HUVEC pretreated with pentraxins were incubated with FHR-5 or FHR-1, then exposed to 5 or 10% NHS, respectively, in Mg^2+^-EGTA containing buffer, to allow only for alternative pathway activation. Different serum concentrations were used because FHR-5 activates the alternative pathway more efficiently compared with FHR-1 (36, 37). Formation of the alternative pathway C3bBb convertase on the necrotic cells was detected with flow cytometry using anti-FB Ab. Recruitment of both FHR-1 and FHR-5 by pentraxins increased the amount of detectable C3bBb on necrotic cells (Figure 10).

## Discussion

Opsonization, i.e., marking of non-self and certain self materials for removal, is an important mechanism to maintain homeostasis. Opsonization is a major function of complement through which elimination of microbes but also potentially dangerous host material, such as disintegrating dying cells and extracellular DNA, is facilitated. In this study we identify the FHR proteins FHR-1 and FHR-5 as modulators of opsonization by their interactions with DNA, dead cells and pentraxins.

The role of FH in complement regulation on the surface of apoptotic and necrotic cells, and in promoting an immunologically silent removal of dead cells was studied by several groups. FH binding to dead cells increases during the apoptotic process and reaches its maximum at the necrotic state (22, 45). Possible ligands for FH on the surface of dead cells were identified as Annexin II, histones and DNA (45). FH binds to necrotic cells through CCP6-8 and CCP19-20, which mediate also the DNA binding of the molecule; besides these domains, CCP8-15 are involved in FH binding to apoptotic cells (45, 55). We found that the two FHR proteins FHR-1 and FHR-5 bound to linearized plasmid and also to isolated human gDNA in a dose-dependent manner (Figure 1). The DNA binding site of FHR-1 was in the C terminus, which can be explained by the 100 and 97% amino acid sequence identity of CCP4 and CCP5 of FHR-1 to the CCP19 and CCP20 of FH, respectively. Because the anti-FH mAb C18, which binds to FH CCP20 and FHR-1 CCP5, completely blocked FHR-1 binding to gDNA, CCP5 appears to be the DNA binding domain in FHR-1 (Figure 2B). Interestingly, the homologous CCP8-9 of FHR-5 did not bind to DNA, but the DNA binding site was localized in CCP3-4 (Figure 2A). This is likely explained by the relatively low sequence similarity of FHR-5 CCP8 and CCP9 to the homologous FH CCP19 and CCP20 (66 and 43% amino acid sequence identity, respectively). The DNA-binding specificity of the two FHRs was confirmed by dNTP preincubation (Figure 2). In addition, we demonstrated that they did not only bind to extracted gDNA but both FHR-1 and FHR-5 associated with NETs and colocalized with DNA (Supplementary Figure 1).

Evidence accumulated in recent years attest to a role of FHR proteins in the modulation of complement activation and its regulation by FH, by enhancing the activity of the alternative pathway (56). Common host ligands of FHR-1 and FHR-5 shared also by FH are, besides the complement fragment C3b, heparin (29, 31), the pentraxins mCRP and PTX3 (31, 36, 37) and, in the case of FHR-5 also malondialdehyde-acetaldehyde epitopes (57). FHR-5 competes with FH for binding to mCRP, PTX3 and malondialdehyde-acetaldehyde epitopes, and enhances complement activation (36, 57). For FHR-1, partial inhibition of FH binding to mCRP was found but complement activation was not significantly enhanced in serum through competition; on the other hand, FHR-1—mCRP interaction directly enhanced both alternative and classical pathway activation *in vitro* by the recruitment of C3b and C1q, respectively (37). Here, we identified DNA as a new ligand for FHR-1 and FHR-5, and showed that both FHRs dose-dependently inhibited FH binding to gDNA and, consequently, inhibited FH cofactor activity on DNA (Figure 3). Both FHR-5 and FHR-1 enhanced complement alternative pathway activation on DNA when added to serum, as measured by the increase in the amount of deposited C3 and FB fragments, but FHR-1 was less efficient in this assay (Figure 4). FHR-5 appears as a more potent competitor of FH and activator of the alternative pathway than FHR-1, because it can use separate binding sites for C3b (the C-terminal domains CCPs 8–9) and other ligands (CCPs 3–4 or 5–7), such as malondialdehyde-acetaldehyde adducts (57), DNA (Figure 2), mCRP (31) and PTX3 (Supplementary Figure 3), whereas FHR-1 ligand binding sites e.g., for both C3b and DNA (but also for pentraxins) are in the C-terminal domains (37). FHR-1 requires a higher density of ligands for similarly high avidity binding as FHR-5; however, gain-of-function mutants resulting in duplication of the dimerization CCPs 1–2 domains strongly increase avidity and FH-competing capacity (39, 43).

It was shown previously, that FHR-5 does not bind or only weakly binds to live cells, such as HUVEC, from NHS, but binds strongly to necrotic HUVEC, also as a recombinant protein (48); FHR-5 binding was mediated by CCPs 5–7 (57). We found variable binding of recombinant FHR-5 to live cells, the weakest binding in the case of ARPE-19 cells and the strongest binding in the case of HUVEC. FHR-5 binding strongly increased in the necrotic state of the cells, and pronounced binding was detected also on early and late apoptotic cells (Figure 5). Although FHR-1 binding from NHS to live HUVEC was observed previously by Western blot analysis (48, 58), we could not detect recombinant FHR-1 binding to live HUVEC, ARPE-19 and Jurkat cells by flow cytometry (Figure 6A). This might be explained by the binding through C3 fragments or other proteins in the case of serum. Similar to FHR-5, the extent of FHR-1 binding was increased when the cells became apoptotic or necrotic (Figure 6). A recent study also found FHR-1 binding to necrotic HUVEC but not to live HUVEC (59). Binding of FHR-1 and FHR-5 to cells depends on available receptors and surface ligands; for example, previously we did demonstrate FHR-1 binding to viable neutrophils via the complement receptor CR3 (60). On necrotic cells, malondialdehyde epitopes represent one of the ligands of FHR-1 and FHR-5, and both FHRs were shown to enhance complement activation when bound to malondialdehyde epitopes *in vitro* (57, 61). The inhibition of FHR-1 and FHR-5 binding to necrotic cells by dNTP and their partial co-localization with DNA suggest that DNA could serve as an additional ligand for both FHRs on necrotic cells (Supplementary Figure 2). FHR-1 and FHR-5 have been linked to glomerular diseases, where pathogenic, gain-of-function variants cause enhanced complement activation (34, 38, 39, 48) [for more details on disease associations see (43)], and the presence of these proteins at sites of tissue damage has been detected (62, 63). Thus, among other serum proteins like FH and C4BP (22), FHR-1 and FHR-5 binding is increased during cell death and may regulate the deposition of complement-derived opsonins on the cell surface. In addition, FHR-1 has recently been shown to associate with necrotic sites in glomeruli and in atherosclerotic plaques and, when bound to necrotic cells, FHR-1 could induce IL-1β release from monocytes, thus having a pro-inflammatory effect (59).

Pentraxins are implicated in inflammatory diseases and the clearance of dead cells, regulate opsonization, and have manifold interactions with the complement system (8, 10–12, 14, 64–67). Both CRP and PTX3 are expressed and upregulated under inflammatory conditions and may deposit locally at the site of tissue damage, also in complement-related diseases such as age-related macular degeneration and atypical hemolytic uremic syndrome (68–72). Purified CRP or CRP from NHS does not bind to live cells but binds weakly to apoptotic cells and more strongly to necrotic cells (21, 22, 73). Recruitment of FH by CRP to dead cells was a seemingly contradictory issue because some studies used pCRP, while others used the modified mCRP form that is generated under inflammatory conditions (21, 22, 50). The three FH family proteins FH, FHR-5, and FHR-1 were indeed shown to interact primarily with mCRP (36, 37, 42, 50, 66, 74, 75), and they also bind to PTX3 (12, 13, 36). Recruitment of FH by CRP limited complement activation at the C3 level and inhibited terminal complement pathway activation and facilitated removal of late apoptotic cells in an anti-inflammatory manner (21, 50). Similar recruitment of FH by PTX3 was described on late apoptotic Jurkat cells and resulted in increased amounts of inactive C3b (iC3b) (12). Recruitment of FHR-1 by mCRP, however, supported alternative pathway activation at the C3 level on the surface of necrotic HUVECs (37). In the present study, we investigated the role of FHR-1 and FHR-5 in the modulation of opsonization of necrotic cells through their interactions with pentraxins. FHR-1 and FHR-5 were able to recruit both mCRP and PTX3 to the necrotic cell surface and *vice versa* (Figure 7); thus, the two pentraxins can collaborate with FHR-1 and FHR-5 on necrotic cells. This was clearly demonstrated by the functional consequence of these interactions, namely, the increase in the deposition of C3 and C4 fragments, and the formation of the alternative pathway C3 convertase on necrotic HUVECs (Figures 8–10). Thus, the cooperation between both FHR-1 and FHR-5 with mCRP and PTX3 considerably influences both classical and alternative complement pathway activation on dead cells and, therefore, the opsonization pattern on the dead cell surface. Whether such a process also occurs on NETs and whether interactions of these FHRs and pentraxins similarly enhance complement activation need to be studied.

Normal serum FH levels vary significantly, ranging 124.4–402 μg/ml (~0.8–2.59 μM) but can be even lower in diseases with FH deficiency (76, 77). FHR-1 serum levels are strongly affected by a common gene deletion; thus, reported average FHR-1 concentrations range from 0 to 122 μg/ml (0–3 μM) (78). However, FHR-1 quantification is controversial, and another recent study reported lower concentrations, 14.64 μg/ml (~185 nM) for FHR-1 homodimers and 5.84 μg/ml (~87 nM) for FHR-1/FHR-2 heterodimers in those having two copies of *CFHR1* (79). For FHR-5, serum levels ranging from 1.66 to 10.1 μg/ml (~155 nM) were reported (31, 79, 80). In tissues, local concentrations may differ and are also influenced by the availability of ligands; increasing ligand density and/or certain FHR-1 and FHR-5 variants with enhanced avidity can result in increased local complement activation, as reviewed in detail elsewhere (43, 56). In our experiments, we applied the studied proteins in concentrations considering the above data, and therefore, we believe that the results are relevant and suggest that, particularly under disease conditions, FHR-1 and FHR-5 can promote complement activation.

FHR proteins were shown to enhance complement activation and C3 deposition on surfaces, including altered host surfaces such as those of necrotic cells (35–37, 40, 48, 57) and tumor cells (81), but also on microbes (33, 82, 83). Here, we showed that FHR-1 and FHR-5 enhance complement activation on necrotic cells via their interactions with mCRP and PTX3. The role of the FHRs is apparently complex: The proximal complement activity, thus opsonization at the C4 and C3 levels, is enhanced, but inflammation and lysis are likely prevented by FH, C4BP, and other regulators (21, 22, 50), as well as through the reported C5 convertase-inhibiting ability of the FHRs (27, 29, 32, 56).

**Figure 11:**
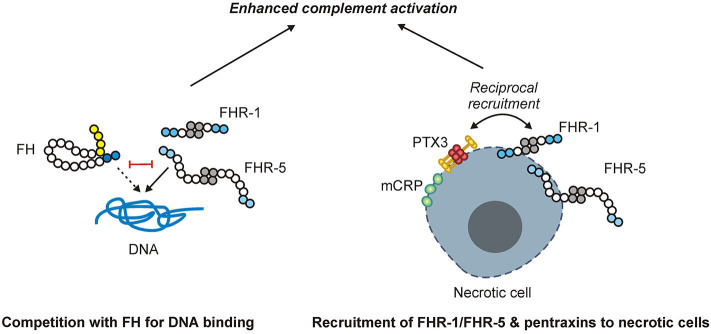
Schematic overview of the main findings. FHR-1 and FHR-5 (shown as homodimers) can bind to DNA and compete with FH for DNA binding, causing enhanced complement activation. While FHR-1 and FHR-5, as well as the pentraxins mCRP and PTX3, can bind to necrotic cells, they also recruit each other to the necrotic cell surface, resulting in enhanced complement activation.

In summary, FHR-1 and FHR-5 are identified and characterized as regulators of complement activation on DNA and dead cells and are shown to activate not only the alternative pathway but also the classical pathway through their interactions with pentraxins (Figure 11). These results lend further evidence for the role of FHR proteins as positive modulators of complement activation and enhancers of opsonization.

## Data Availability Statement

The raw data supporting the conclusions of this article will be made available by the authors, without undue reservation.

## Ethics Statement

The studies involving human participants were approved by the respective national authority (TUKEB ETT, permission number 838/PI/12). The participants provided their informed consent.

## Author's Note

Parts of this work were presented at the 16th European Meeting on Complement in Human Disease, September 8–12, 2017, Copenhagen, Denmark (*Mol. Immunol*. 2017, 89:140–141).

## Author Contributions

MJ initiated and supervised the study. ÉK, BU, and MJ designed the experiments. ÉK, AP, MC, ÁC, and BU cloned, expressed, and purified recombinant proteins and performed ligand binding assays, competition assays, and complement activation assays. ÉK, AS, DH, and BU performed cellular assays. ÉK and MJ wrote the manuscript with the help of the other authors. All authors discussed the data and revised and approved the manuscript.

## Conflict of Interest

The authors declare that the research was conducted in the absence of any commercial or financial relationships that could be construed as a potential conflict of interest.

## Abbreviations

BSA: bovine serum albumin
CCP: complement control protein domain
CRP: C-reactive protein
DPBS: Dulbecco's phosphate-buffered saline
FB: factor B
FD: factor D
FH: factor H
FHR: factor H-related
FHR-1: factor H-related protein 1
FHR-4: factor H-related protein 4
FHR-5: factor H-related protein 5
FI: factor I
FP: factor P
gDNA: genomic DNA
HSA: human serum albumin
mCRP: modified monomeric form of CRP
NET: neutrophil extracellular trap
NHS: normal human serum
pCRP: native pentameric form of CRP
PTX3: pentraxin 3.

## References

[1] Martin M Blom AM. Complement in removal of the dead - balancing inflammation. Immunol Rev. (2016) 274:218–32. 10.1111/imr.12462 27782329

[2] Ricklin D Hajishengallis G Yang K Lambris JD. Complement: a key system for immune surveillance and homeostasis. Nat Immunol. (2010) 11:785–97. 10.1038/ni.1923 20720586 PMC2924908

[3] Gaipl US Franz S Voll RE Sheriff A Kalden JR Herrmann M. Defects in the disposal of dying cells lead to autoimmunity. Curr Rheumatol Rep. (2004) 6:401–7. 10.1007/s11926-004-0016-1 15527698

[4] Herrmann M Voll RE Zoller OM Hagenhofer M Ponner BB Kalden JR. Impaired phagocytosis of apoptotic cell material by monocyte-derived macrophages from patients with systemic lupus erythematosus. Arthritis Rheum. (1998) 41:1241–50. 10.1002/1529-0131(199807)41:7>1241::AID-ART15<3.0.CO;2-H 9663482

[5] Leers MP Bjorklund V Bjorklund B Jornvall H Nap M. An immunohistochemical study of the clearance of apoptotic cellular fragments. Cell Mol Life Sci. (2002) 59:1358–65. 10.1007/s00018-002-8513-8 12363038 PMC11337528

[6] Radic M Herrmann M van der Vlag J Rekvig OP. Regulatory and pathogenetic mechanisms of autoantibodies in SLE. Autoimmunity. (2011) 44:349–56. 10.3109/08916934.2010.536794 21231891

[7] Gardai SJ Bratton DL Ogden CA Henson PM. Recognition ligands on apoptotic cells: a perspective. J Leukoc Biol. (2006) 79:896–903. 10.1189/jlb.1005550 16641135

[8] Nauta AJ Daha MR van Kooten C Roos A. Recognition and clearance of apoptotic cells: a role for complement and pentraxins. Trends Immunol. (2003) 24:148–54. 10.1016/S1471-4906(03)00030-9 12615211

[9] Ogden CA deCathelineau A Hoffmann PR Bratton D Ghebrehiwet B Fadok VA. C1q and mannose binding lectin engagement of cell surface calreticulin and CD91 initiates macropinocytosis and uptake of apoptotic cells. J Exp Med. (2001) 194:781–95. 10.1084/jem.194.6.781 11560994 PMC2195958

[10] Mantovani A Garlanda C Doni A Bottazzi B. Pentraxins in innate immunity: from C-reactive protein to the long pentraxin PTX3. J Clin Immunol. (2008) 28:1–13. 10.1007/s10875-007-9126-7 17828584

[11] Braunschweig A Jozsi M. Human pentraxin 3 binds to the complement regulator c4b-binding protein. PLoS ONE. (2011) 6:e23991. 10.1371/journal.pone.0023991 21915248 PMC3161823

[12] Deban L Jarva H Lehtinen MJ Bottazzi B Bastone A Doni A. Binding of the long pentraxin PTX3 to factor H: interacting domains and function in the regulation of complement activation. J Immunol. (2008) 181:8433–40. 10.4049/jimmunol.181.12.8433 19050261

[13] Kopp A Strobel S Tortajada A Rodriguez de Cordoba S Sanchez-Corral P Prohaszka Z. Atypical hemolytic uremic syndrome-associated variants and autoantibodies impair binding of factor h and factor h-related protein 1 to pentraxin 3. J Immunol. (2012) 189:1858–67. 10.4049/jimmunol.1200357 22786770

[14] Sjoberg AP Trouw LA McGrath FD Hack CE Blom AM. Regulation of complement activation by C-reactive protein: targeting of the inhibitory activity of C4b-binding protein. J Immunol. (2006) 176:7612–20. 10.4049/jimmunol.176.12.7612 16751408

[15] Kemper C Mitchell LM Zhang L Hourcade DE. The complement protein properdin binds apoptotic T cells and promotes complement activation and phagocytosis. Proc Natl Acad Sci USA. (2008) 105:9023–8. 10.1073/pnas.0801015105 18579773 PMC2449358

[16] Xu W Berger SP Trouw LA de Boer HC Schlagwein N Mutsaers C. Properdin binds to late apoptotic and necrotic cells independently of C3b and regulates alternative pathway complement activation. J Immunol. (2008) 180:7613–21. 10.4049/jimmunol.180.11.7613 18490764

[17] Blatt AZ Pathan S Ferreira VP. Properdin: a tightly regulated critical inflammatory modulator. Immunol Rev. (2016) 274:172–90. 10.1111/imr.12466 27782331 PMC5096056

[18] Kemper C Atkinson JP Hourcade DE. Properdin: emerging roles of a pattern-recognition molecule. Annu Rev Immunol. (2010) 28:131–55. 10.1146/annurev-immunol-030409-101250 19947883

[19] Pillemer L Blum L Lepow IH Ross OA Todd EW Wardlaw AC. The properdin system and immunity. I. Demonstration and isolation of a new serum protein, properdin, and its role in immune phenomena. Science. (1954) 120:279–85. 10.1126/science.120.3112.279 13186838

[20] Mevorach D Mascarenhas JO Gershov D Elkon KB. Complement-dependent clearance of apoptotic cells by human macrophages. J Exp Med. (1998) 188:2313–20. 10.1084/jem.188.12.2313 9858517 PMC2212421

[21] Gershov D Kim S Brot N Elkon KB. C-Reactive protein binds to apoptotic cells, protects the cells from assembly of the terminal complement components, and sustains an antiinflammatory innate immune response: implications for systemic autoimmunity. J Exp Med. (2000) 192:1353–64. 10.1084/jem.192.9.1353 11067883 PMC2193350

[22] Trouw LA Bengtsson AA Gelderman KA Dahlback B Sturfelt G Blom AM. C4b-binding protein and factor H compensate for the loss of membrane-bound complement inhibitors to protect apoptotic cells against excessive complement attack. J Biol Chem. (2007) 282:28540–8. 10.1074/jbc.M704354200 17699521

[23] Trouw LA Nilsson SC Goncalves I Landberg G Blom AM. C4b-binding protein binds to necrotic cells and DNA, limiting DNA release and inhibiting complement activation. J Exp Med. (2005) 201:1937–48. 10.1084/jem.20050189 15967823 PMC2212022

[24] Jozsi M Tortajada A Uzonyi B Goicoechea de Jorge E Rodriguez de Cordoba S. Factor H-related proteins determine complement-activating surfaces. Trends Immunol. (2015) 36:374–84. 10.1016/j.it.2015.04.008 25979655

[25] Medjeral-Thomas N Pickering MC. The complement factor H-related proteins. Immunol Rev. (2016) 274:191–201. 10.1111/imr.12477 27782332

[26] Skerka C Chen Q Fremeaux-Bacchi V Roumenina LT. Complement factor H related proteins (CFHRs). Mol Immunol. (2013) 56:170–80. 10.1016/j.molimm.2013.06.001 23830046

[27] Eberhardt HU Buhlmann D Hortschansky P Chen Q Bohm S Kemper MJ. Human factor H-related protein 2 (CFHR2) regulates complement activation. PLoS ONE. (2013) 8:e78617. 10.1371/journal.pone.0078617 24260121 PMC3832495

[28] Fritsche LG Lauer N Hartmann A Stippa S Keilhauer CN Oppermann M. An imbalance of human complement regulatory proteins CFHR1, CFHR3 and factor H influences risk for age-related macular degeneration (AMD). Hum Mol Genet. (2010) 19:4694–704. 10.1093/hmg/ddq399 20843825

[29] Heinen S Hartmann A Lauer N Wiehl U Dahse HM Schirmer S. Factor H-related protein 1 (CFHR-1) inhibits complement C5 convertase activity and terminal complex formation. Blood. (2009) 114:2439–47. 10.1182/blood-2009-02-205641 19528535

[30] Hellwage J Jokiranta TS Koistinen V Vaarala O Meri S Zipfel PF. Functional properties of complement factor H-related proteins FHR-3 and FHR-4: binding to the C3d region of C3b and differential regulation by heparin. FEBS Lett. (1999) 462:345–52. 10.1016/S0014-5793(99)01554-9 10622723

[31] McRae JL Duthy TG Griggs KM Ormsby RJ Cowan PJ Cromer BA. Human factor H-related protein 5 has cofactor activity, inhibits C3 convertase activity, binds heparin and C-reactive protein, and associates with lipoprotein. J Immunol. (2005) 174:6250–6. 10.4049/jimmunol.174.10.6250 15879123

[32] Zwarthoff SA Berends ETM Mol S Ruyken M Aerts PC Jozsi M. Functional characterization of alternative and classical pathway C3/C5 convertase activity and inhibition using purified models. Front Immunol. (2018) 9:1691. 10.3389/fimmu.2018.01691 30083158 PMC6064732

[33] Caesar JJ Lavender H Ward PN Exley RM Eaton J Chittock E Competition between antagonistic complement factors for a single protein on N. meningitidis rules disease susceptibility. Elife. (2014) 3:e04008 10.7554/eLife.04008 PMC4273445 25534642

[34] Chen Q Wiesener M Eberhardt HU Hartmann A Uzonyi B Kirschfink M. Complement factor H-related hybrid protein deregulates complement in dense deposit disease. J Clin Invest. (2014) 124:145–55. 10.1172/JCI71866 24334459 PMC3871254

[35] Cserhalmi M Csincsi AI Mezei Z Kopp A Hebecker M Uzonyi B. The murine factor H-related protein FHR-B promotes complement activation. Front Immunol. (2017) 8:1145. 10.3389/fimmu.2017.01145 28974948 PMC5610720

[36] Csincsi AI Kopp A Zoldi M Banlaki Z Uzonyi B Hebecker M. Factor H-related protein 5 interacts with pentraxin 3 and the extracellular matrix and modulates complement activation. J Immunol. (2015) 194:4963–73. 10.4049/jimmunol.1403121 25855355 PMC4416742

[37] Csincsi AI Szabo Z Banlaki Z Uzonyi B Cserhalmi M Karpati E. FHR-1 binds to C-reactive protein and enhances rather than inhibits complement activation. J Immunol. (2017) 199:292–303. 10.4049/jimmunol.1600483 28533443

[38] Goicoechea de Jorge E Caesar JJ Malik TH Patel M Colledge M Johnson S. Dimerization of complement factor H-related proteins modulates complement activation *in vivo*. Proc Natl Acad Sci USA. (2013) 110:4685–90. 10.1073/pnas.1219260110 23487775 PMC3606973

[39] Tortajada A Yebenes H Abarrategui-Garrido C Anter J Garcia-Fernandez JM Martinez-Barricarte R. C3 glomerulopathy-associated CFHR1 mutation alters FHR oligomerization and complement regulation. J Clin Invest. (2013) 123:2434–46. 10.1172/JCI68280 23728178 PMC3668852

[40] Hebecker M Jozsi M. Factor H-related protein 4 activates complement by serving as a platform for the assembly of alternative pathway C3 convertase via its interaction with C3b protein. J Biol Chem. (2012) 287:19528–36. 10.1074/jbc.M112.364471 22518841 PMC3365989

[41] Hebecker M Okemefuna AI Perkins SJ Mihlan M Huber-Lang M Jozsi M. Molecular basis of C-reactive protein binding and modulation of complement activation by factor H-related protein 4. Mol Immunol. (2010) 47:1347–55. 10.1016/j.molimm.2009.12.005 20042240

[42] Mihlan M Hebecker M Dahse HM Halbich S Huber-Lang M Dahse R. Human complement factor H-related protein 4 binds and recruits native pentameric C-reactive protein to necrotic cells. Mol Immunol. (2009) 46:335–44. 10.1016/j.molimm.2008.10.029 19084272

[43] Sanchez-Corral P Pouw RB Lopez-Trascasa M Jozsi M. Self-damage caused by dysregulation of the complement alternative pathway: relevance of the factor H protein family. Front Immunol. (2018) 9:1607. 10.3389/fimmu.2018.01607 30050540 PMC6052053

[44] Smith RJH Appel GB Blom AM Cook HT D'Agati VD Fakhouri F. C3 glomerulopathy - understanding a rare complement-driven renal disease. Nat Rev Nephrol. (2019) 15:129–43. 10.1038/s41581-018-0107-2 30692664 PMC6876298

[45] Leffler J Herbert AP Norstrom E Schmidt CQ Barlow PN Blom AM. Annexin-II, DNA, and histones serve as factor H ligands on the surface of apoptotic cells. J Biol Chem. (2010) 285:3766–76. 10.1074/jbc.M109.045427 19951950 PMC2823518

[46] Martin M Leffler J Smolag KI Mytych J Bjork A Chaves LD. Factor H uptake regulates intracellular C3 activation during apoptosis and decreases the inflammatory potential of nucleosomes. Cell Death Differ. (2016) 23:903–11. 10.1038/cdd.2015.164 26768663 PMC4832108

[47] Halder LD Abdelfatah MA Jo EA Jacobsen ID Westermann M Beyersdorf N. Factor H binds to extracellular DNA traps released from human blood monocytes in response to candida albicans. Front Immunol. (2017) 7:671. 10.3389/fimmu.2016.00671 28133459 PMC5233719

[48] Chen Q Manzke M Hartmann A Buttner M Amann K Pauly D. Complement factor H-related 5-hybrid proteins anchor properdin and activate complement at self-surfaces. J Am Soc Nephrol. (2016) 27:1413–25. 10.1681/ASN.2015020212 26432903 PMC4849819

[49] Kuhn S Zipfel PF. The baculovirus expression vector pBSV-8His directs secretion of histidine-tagged proteins. Gene. (1995) 162:225–9. 10.1016/0378-1119(95)00360-I 7557433

[50] Mihlan M Stippa S Jozsi M Zipfel PF. Monomeric CRP contributes to complement control in fluid phase and on cellular surfaces and increases phagocytosis by recruiting factor H. Cell Death Differ. (2009) 16:1630–40. 10.1038/cdd.2009.103 19680263

[51] Schneider AE Sandor N Karpati E Jozsi M. Complement factor H modulates the activation of human neutrophil granulocytes and the generation of neutrophil extracellular traps. Mol Immunol. (2016) 72:37–48. 10.1016/j.molimm.2016.02.011 26938503

[52] Bhattacharjee A Reuter S Trojnar E Kolodziejczyk R Seeberger H Hyvarinen S. The major autoantibody epitope on factor H in atypical hemolytic uremic syndrome is structurally different from its homologous site in factor H-related protein 1, supporting a novel model for induction of autoimmunity in this disease. J Biol Chem. (2015) 290:9500–10. 10.1074/jbc.M114.630871 25659429 PMC4392255

[53] Oppermann M Manuelian T Jozsi M Brandt E Jokiranta TS Heinen S. The C-terminus of complement regulator Factor H mediates target recognition: evidence for a compact conformation of the native protein. Clin Exp Immunol. (2006) 144:342–52. 10.1111/j.1365-2249.2006.03071.x 16634809 PMC1809651

[54] Parker H Dragunow M Hampton MB Kettle AJ Winterbourn CC. Requirements for NADPH oxidase and myeloperoxidase in neutrophil extracellular trap formation differ depending on the stimulus. J Leukoc Biol. (2012) 92:841–9. 10.1189/jlb.1211601 22802447

[55] Sjoberg AP Trouw LA Clark SJ Sjolander J Heinegard D Sim RB. The factor H variant associated with age-related macular degeneration (His-384) and the non-disease-associated form bind differentially to C-reactive protein, fibromodulin, DNA, and necrotic cells. J Biol Chem. (2007) 282:10894–900. 10.1074/jbc.M610256200 17293598

[56] Cserhalmi M Papp A Brandus B Uzonyi B Jozsi M. Regulation of regulators: role of the complement factor H-related proteins. Semin Immunol. (2019) 45:101341. 10.1016/j.smim.2019.101341 31757608

[57] Rudnick RB Chen Q Stea ED Hartmann A Papac-Milicevic N Person F. FHR5 binds to laminins, uses separate C3b and surface-binding sites, and activates complement on malondialdehyde-acetaldehyde surfaces. J Immunol. (2018) 200:2280–90. 10.4049/jimmunol.1701641 29483359

[58] Strobel S Abarrategui-Garrido C Fariza-Requejo E Seeberger H Sanchez-Corral P Jozsi M. Factor H-related protein 1 neutralizes anti-factor H autoantibodies in autoimmune hemolytic uremic syndrome. Kidney Int. (2011) 80:397–404. 10.1038/ki.2011.152 21677636

[59] Irmscher S Brix SR Zipfel SLH Halder LD Mutluturk S Wulf S. Serum FHR1 binding to necrotic-type cells activates monocytic inflammasome and marks necrotic sites in vasculopathies. Nat Commun. (2019) 10:2961. 10.1038/s41467-019-10766-0 31273197 PMC6609651

[60] Losse J Zipfel PF Jozsi M. Factor H and factor H-related protein 1 bind to human neutrophils via complement receptor 3, mediate attachment to Candida albicans, and enhance neutrophil antimicrobial activity. J Immunol. (2010) 184:912–21. 10.4049/jimmunol.0901702 20008295

[61] Alic L Papac-Milicevic N Czamara D Rudnick RB Ozsvar-Kozma M Hartmann A. A genome-wide association study identifies key modulators of complement factor H binding to malondialdehyde-epitopes. Proc Natl Acad Sci USA. (2020) 117:9942–51. 10.1073/pnas.1913970117 32321835 PMC7211993

[62] McRae JL Cowan PJ Power DA Mitchelhill KI Kemp BE Morgan BP. Human factor H-related protein 5 (FHR-5). A new complement-associated protein. J Biol Chem. (2001) 276:6747–54. 10.1074/jbc.M007495200 11058592

[63] Sethi S Gamez JD Vrana JA Theis JD Bergen HR III Zipfel PF. Glomeruli of dense deposit disease contain components of the alternative and terminal complement pathway. Kidney Int. (2009) 75:952–60. 10.1038/ki.2008.657 19177158 PMC2738640

[64] Bottazzi B Doni A Garlanda C Mantovani A. An integrated view of humoral innate immunity: pentraxins as a paradigm. Annu Rev Immunol. (2010) 28:157–83. 10.1146/annurev-immunol-030409-101305 19968561

[65] Haapasalo K Meri S. Regulation of the complement system by pentraxins. Front Immunol. (2019) 10:1750. 10.3389/fimmu.2019.01750 31428091 PMC6688104

[66] Jarva H Jokiranta TS Hellwage J Zipfel PF Meri S. Regulation of complement activation by C-reactive protein: targeting the complement inhibitory activity of factor H by an interaction with short consensus repeat domains 7 and 8–11. J Immunol. (1999) 163:3957–62. 10490997

[67] Trouw LA Blom AM Gasque P. Role of complement and complement regulators in the removal of apoptotic cells. Mol Immunol. (2008) 45:1199–207. 10.1016/j.molimm.2007.09.008 17961651

[68] Deban L Jaillon S Garlanda C Bottazzi B Mantovani A. Pentraxins in innate immunity: lessons from PTX3. Cell Tissue Res. (2011) 343:237–49. 10.1007/s00441-010-1018-0 20683616

[69] Laine M Jarva H Seitsonen S Haapasalo K Lehtinen MJ Lindeman N. Y402H polymorphism of complement factor H affects binding affinity to C-reactive protein. J Immunol. (2007) 178:3831–6. 10.4049/jimmunol.178.6.3831 17339482 PMC4853917

[70] Pepys MB Hirschfield GM. C-reactive protein: a critical update. J Clin Invest. (2003) 111:1805–12. 10.1172/JCI200318921 12813013 PMC161431

[71] Trojnar E Jozsi M Szabo Z Reti M Farkas P Kelen K. Elevated systemic pentraxin-3 is associated with complement consumption in the acute phase of thrombotic microangiopathies. Front Immunol. (2019) 10:240. 10.3389/fimmu.2019.00240 30858847 PMC6397851

[72] Woo JM Kwon MY Shin DY Kang YH Hwang N Chung SW. Human retinal pigment epithelial cells express the long pentraxin PTX3. Mol Vis. (2013) 19:303–10. 23401658 PMC3566900

[73] Hart SP Alexander KM MacCall SM Dransfield I. C-reactive protein does not opsonize early apoptotic human neutrophils, but binds only membrane-permeable late apoptotic cells and has no effect on their phagocytosis by macrophages. J Inflamm. (2005) 2:5. 10.1186/1476-9255-2-5 15927062 PMC1177984

[74] Hakobyan S Harris CL van den Berg CW Fernandez-Alonso MC de Jorge EG de Cordoba SR. Complement factor H binds to denatured rather than to native pentameric C-reactive protein. J Biol Chem. (2008) 283:30451–60. 10.1074/jbc.M803648200 18786923 PMC2662140

[75] Mold C Gewurz H Du Clos TW. Regulation of complement activation by C-reactive protein. Immunopharmacology. (1999) 42:23–30. 10.1016/S0162-3109(99)00007-7 10408362

[76] Dragon-Durey MA Fremeaux-Bacchi V Loirat C Blouin J Niaudet P Deschenes G Heterozygous and homozygous factor h deficiencies associated with hemolytic uremic syndrome or membranoproliferative glomerulonephritis: report and genetic analysis of 16 cases. J Am Soc Nephrol. (2004) 15:787–95. 10.1097/01.ASN.0000115702.28859.A7 14978182

[77] Hakobyan S Tortajada A Harris CL de Cordoba SR Morgan BP. Variant-specific quantification of factor H in plasma identifies null alleles associated with atypical hemolytic uremic syndrome. Kidney Int. (2010) 78:782–8. 10.1038/ki.2010.275 20703214 PMC3252682

[78] Tortajada A Gutierrez E Goicoechea de Jorge E Anter J Segarra A Espinosa M. Elevated factor H-related protein 1 and factor H pathogenic variants decrease complement regulation in IgA nephropathy. Kidney Int. (2017) 92:953–63. 10.1016/j.kint.2017.03.041 28637589

[79] van Beek AE Pouw RB Brouwer MC van Mierlo G Geissler J Ooijevaar-de Heer P. Factor H-related (FHR)-1 and FHR-2 form homo- and heterodimers, while FHR-5 circulates only as homodimer in human plasma. Front Immunol. (2017) 8:1328. 10.3389/fimmu.2017.01328 29093712 PMC5651247

[80] Vernon KA Goicoechea de Jorge E Hall AE Fremeaux-Bacchi V Aitman TJ Cook HT Acute presentation and persistent glomerulonephritis following streptococcal infection in a patient with heterozygous complement factor H-related protein 5 deficiency. Am J Kidney Dis. (2012) 60:121–5. 10.1053/j.ajkd.2012.02.329 22503529 PMC3382710

[81] Seguin-Devaux C Plesseria JM Verschueren C Masquelier C Iserentant G Fullana M. FHR4-based immunoconjugates direct complement-dependent cytotoxicity and phagocytosis towards HER2-positive cancer cells. Mol Oncol. (2019) 13:2531–53. 10.1002/1878-0261.12554 31365168 PMC6887587

[82] Jozsi M. Factor H family proteins in complement evasion of microorganisms. Front Immunol. (2017) 8:571. 10.3389/fimmu.2017.00571 28572805 PMC5435753

[83] Reiss T Rosa TFA Blaesius K Bobbert RP Zipfel PF Skerka C. Cutting edge: FHR-1 binding impairs factor H-mediated complement evasion by the malaria parasite *Plasmodium falciparum*. J Immunol. (2018) 201:3497–502. 10.4049/jimmunol.1800662 30455399

