# Supplementary material for: Interaction of the Factor H Family Proteins FHR-1 and FHR-5 With DNA and Dead Cells: Implications for the Regulation of Complement Activation and Opsonization
Source: Front Immunol. 2020 Jul 16;11:1297. doi: 10.3389/fimmu.2020.01297 (PMC7378360; doi:10.3389/fimmu.2020.01297)
Supplement: Supplementary file 1 [file Data_Sheet_1.PDF]

Kárpáti et al. 2020 – Supplementary Figures

Supplemental Fig. S1.

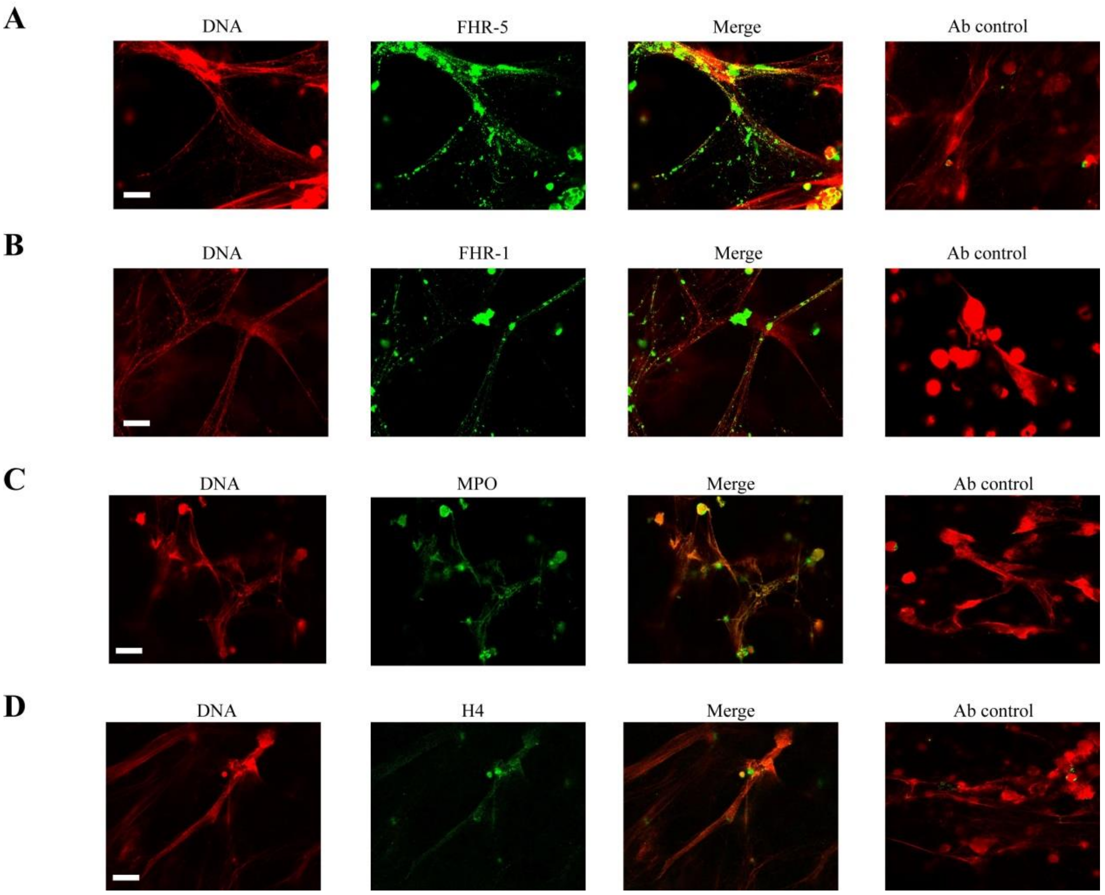

Figure S1. FHR-5 and FHR-1 bind to neutrophil extracellular traps (NETs). Neutrophils were stimulated with 100 nM PMA for 3 hours and NET formation was visualized by DNA staining with Sytox Orange (red). Samples were then incubated with 300 nM FHR-5 (A) or 300 nM FHR-1 (B). Binding was detected using anti-FHR-5 or anti-FH and the corresponding Alexa488-conjugated secondary Ab (green). Representative images from 3 independent experiments are shown. To confirm NET formation, samples were stained for myeloperoxidase (C, MPO) and citrullinated histones (D, H4). Original scale bars, 20 µm. Images of a representative experiment out of three performed are shown.

Supplemental Fig. S2

A

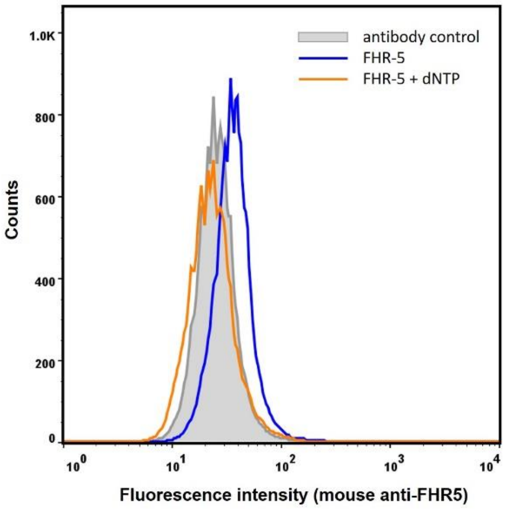

B

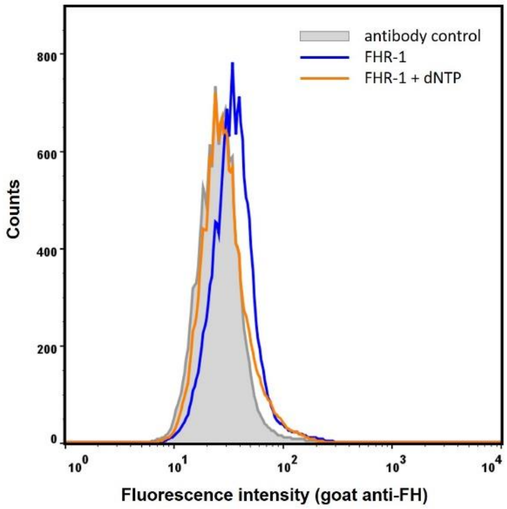

C

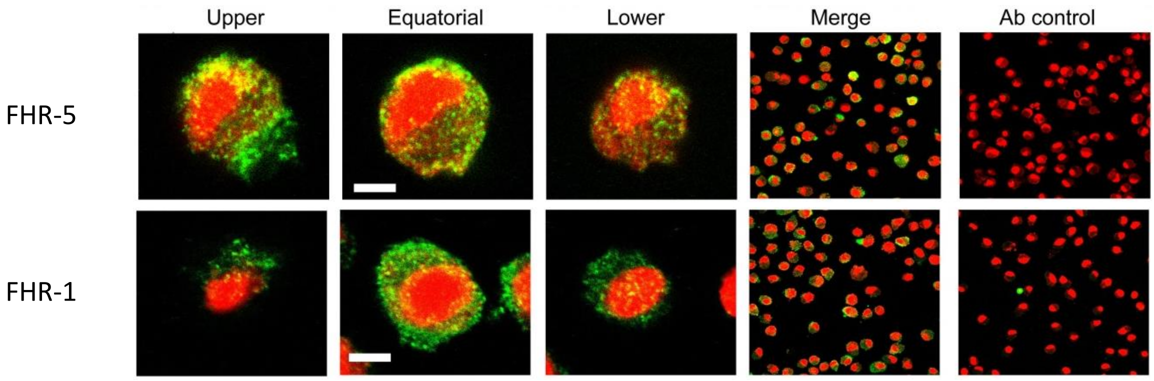

Figure S2. Role of DNA in binding of FHR-5 and FHR-1 to necrotic cells. (A) FHR-5 (75 nM final concentration) and (B) FHR-1 (300 nM final concentration) were incubated in DPBS containing Ca^{2+} and Mg^{2+} with or without 12.5 mM dNTP for 30 min at 22°C. The mixture was added to 5×10^5 necrotic Jurkat cells and incubated for 30 min at 22°C. Cells were then washed and labelled with goat anti-FH to detect FHR-1 binding or mouse anti-FHR-5 to detect FHR-5 binding, followed by the corresponding Alexa488-labelled secondary antibodies, and analyzed by flow cytometry. (C) Representative confocal images obtained with confocal laser scanning microscopy showing the localization of FHR-5 or FHR-1 on necrotic HUVEC. Cells were incubated with 300 nM FHR-5 or FHR-1, followed by incubation with anti-FHR-5 and anti-FH antibodies, respectively, and the corresponding Alexa488-labelled secondary antibodies (both green fluorescence). Nuclei were stained with propidium-iodide (red). Antibody binding in the absence of FHR-5 or FHR-1 is shown as negative control. Original scale bars, 5 µm (zoomed images). Representative results from two experiments are shown.

Supplemental Fig. S3.

A

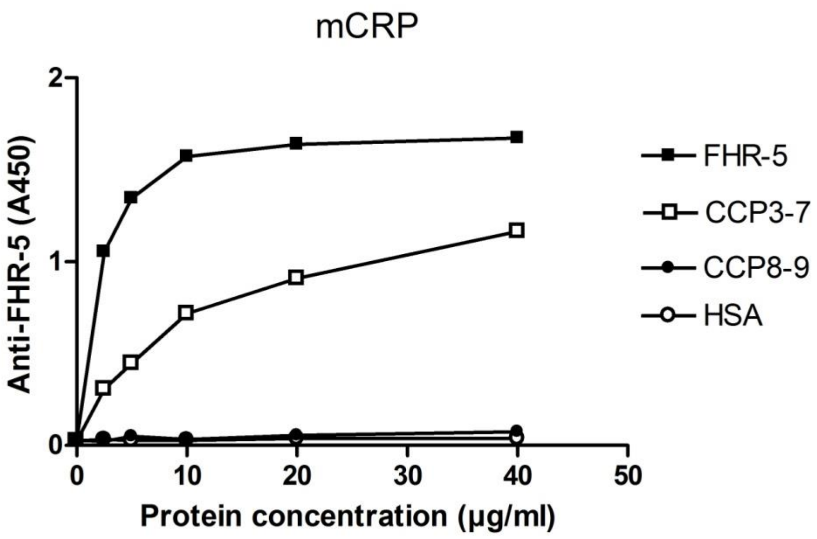

B

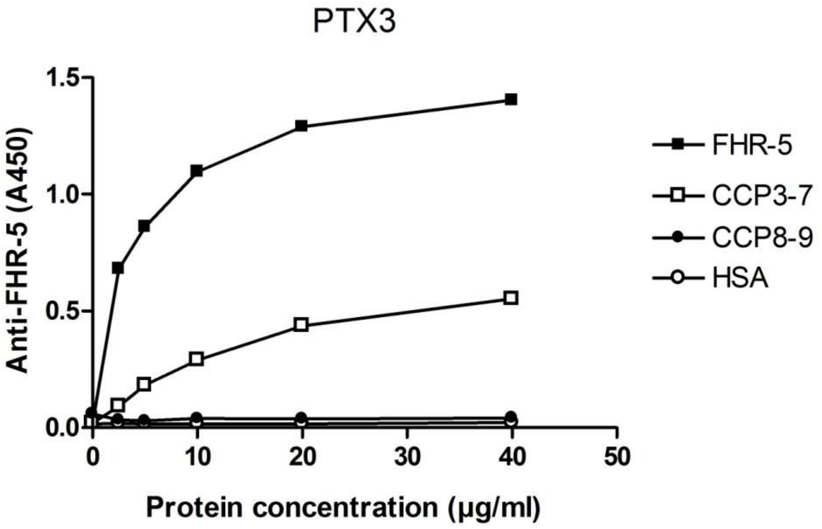

Figure S3. Determination of pentraxin binding site within FHR-5. ELISA was carried out to determine the mCRP (A) and PTX3 (B) binding site in FHR-5. (A) mCRP and (B) PTX3 were immobilized in 10 µg/ml in microplate wells and, after blocking and washing, incubated with serial dilutions of recombinant FHR-5, the FHR-5 fragments CCP3-7 and CCP8-9, and human serum albumin (HSA) as a negative control. Binding was detected with polyclonal anti-FHR-5 Ab. A representative experiment is shown.
